# An effector of phosphatidylinositol 3-kinase activity promotes *Rickettsia rickettsii* virulence by enhancing autophagy

**DOI:** 10.1128/mbio.02284-25

**Published:** 2025-09-22

**Authors:** Dan Huang, Xuan OuYang, Zhihan Peng, Dong Chen, Lei Song, Zhao-Qing Luo

**Affiliations:** 1 Department of Respiratory Medicine, Center of Infectious Diseases and Pathogen Biology, Key Laboratory of Organ Regeneration and Transplantation of the Ministry of Education, State Key Laboratory for Diagnosis and Treatment of Severe Zoonotic Infectious Diseases, The First Hospital of Jilin University 117971, Changchun, China; 2 State Key Laboratory of Pathogen and Biosecurity, Academy of Military Medical Sciences 71040, Beijing, China; University of California, Berkeley, Berkeley, California, USA

**Keywords:** *R. rickettsii*, PikA, T4SS, kinase, phosphoinositides metabolism, autophagy

## Abstract

To establish an intracellular niche conductive to growth, some bacterial pathogens deliver virulence factors that modulate phosphoinositides (PIPs) metabolism. PIPs are a family of lipids involved in signaling that regulates key cellular processes. *Rickettsia rickettsii*, the etiological agent of Rocky Mountain spotted fever, codes for a type IV secretion system (T4SS), but the mechanism by which its secreted substrates contribute to virulence remains largely unclear. Here, we found that the T4SS effector PikA of *R. rickettsii* is a phosphatidylinositol 3-kinase (PI3K) that converts phosphatidylinositol (PI) into phosphatidylinositol 3-phosphate (PI3P). This conversion leads to PI3P accumulation at phagophore assembly sites, promoting autophagosome formation through kinase activity-dependent interaction with Beclin 1. The effects of PikA can be suppressed by the PI3P-specific phosphatase Myotubularin. Furthermore, the expression of Myotubularin suppressed intracellular *R. rickettsii* replication, indicating that autophagy induced by elevated PI3P is beneficial for bacterial virulence. Our findings establish that PikA modulates host PI metabolism via its PI3K activity to promote *R. rickettsii* intracellular growth by inducing autophagy.

**IMPORTANCE:**

The phosphatidylinositol derivative PI3P is a key second messenger that regulates multiple cellular processes, particularly membrane trafficking and autophagy. We report here that PikA, a T4SS substrate of *R. rickettsii*, functions as a PI-3 kinase that catalyzes the production of PI3P to promote autophagy influx. PikA achieves this by recruiting Beclin 1 through direct protein-protein interactions. The expression of the dual-specific PI phosphatase Myotubularin counteracted the effects of PikA and inhibited intracellular *R*. *rickettsii* replication. Our results reveal that the modulation of PI metabolism by a bacterial PI-3 kinase is critical for *R*. *rickettsii* virulence, and this pathway may provide potential target for the development of therapeutics against infections caused by this pathogen.

## INTRODUCTION

Members of the *Rickettsia* genus are Gram-negative obligate intracellular bacterial pathogens which are the causative agents of rickettsioses (1). These pathogens infect a diverse array of hosts, including arthropods and mammals. Within these hosts, pathogenic rickettsial species preferentially target endothelial cells, macrophages, and monocytes (2, 3). Blood-feeding arthropods serve as primary vectors that transmit these pathogenic bacteria to humans and other mammals such as mice (4).

Rickettsiae are categorized into four groups: the typhi group (TG), the spotted fever group (SFG), the transitional group (TRG), and the ancestral group (AG) (5, 6). Both the SFG and TG contain notorious etiological agents that have historically plagued humans and continue to reemerge globally (7). Notably, *R. rickettsii* and *R. conorii*, which belong to the SFG, are the etiological agent of Rocky Mountain spotted fever (RMSF) (8) and Mediterranean spotted fever (MSF) (9), respectively. Additionally, *R. typhi* and *R. prowazekii* from the TG are responsible for endemic typhus (10) and epidemic typhus (11), respectively. Spotted fever rickettsioses are associated with severe clinical symptoms and potentially fatal when not appropriately treated, which increasingly threaten human health due to high incidence of infection (12). The absence of effective vaccines further exacerbates these threats. Thus, a better understanding of the virulence mechanism of these pathogens may provide critical insights for the development of improved diagnosis, prevention, and treatment for infections caused by these bacteria.

Rickettsiae belonging to the SFG deploy multiple surface cell antigens (Scas) to facilitate invasion of endothelial cells. This process involves the interaction of Scas with specific host cell receptors, which activates signaling cascades that ultimately lead to phagocytosis (13). Most Scas, with the exception of Sca4 and Sca9, are classified as autotransporters, also known as Sec-dependent type V secretion systems (T5SS) (14). Among them, Sca5, also referred to as OmpB, facilitates adhesion and invasion of non-phagocytic cells by binding to the receptor Ku70, a subunit of DNA-dependent protein kinase (15). This interaction induces actin polymerization at the bacterial foci via clathrin- and caveolin-dependent endocytic events (16, 17). *Rickettsia* spp. also employ multiple functionally redundant proteins, including Sca0 (OmpA) (18–20), Sca1 (21), Sca2 (22), and Adr2 (23) to enhance their infection.

Bacteria-induced actin polymerization and cytoskeleton rearrangement are crucial events in virulence (24, 25). For instance, the intracellular motility of *Listeria monocytogenes* and *Shigella flexneri* is facilitated by the surface proteins ActA and VirG/IcsA, respectively, both of which stimulate the activity of the Arp2/3 complex (26, 27). Similarly, the surface protein RickA of *R. conorii* is a bacterial activator of actin nucleation which relies on the Arp2/3 complex to initiate actin polymerization (28, 29). The Sca2 of SFG *Rickettsia* species contains WASP homology 2 (WH2) motifs flanked by two proline-rich domains (PRDs), which are similar to the formin homology 1 (FH1) domains of formins, and manipulates host cell movement by hijacking actin polymerization (22, 30, 31).

It is interesting to note that homologs of RickA and Sca2 are absent in *R. prowazekii* and *R. typhi*. Recent studies determined that TG rickettsiae have evolved alternative strategies to exploit host phosphoinositide signaling for invasion. For example, the guanine nucleotide exchange factor (GEF) RaIF promotes *R. typhi* entry by activating Arf6, which subsequently activates phosphatidylinositol 4-phosphate 5-kinase (PIP5K) to produce phosphatidylinositol 4,5-bisphosphate (PI (4, 5)P_2_), a molecule long known to induce endocytosis (32–34). PI (4, 5)P_2_ also recruits proteins important for regulation of actin dynamics, such as Cdc42 and Rac1 (35). More recently, the T4SS effector Risk1 of *R. typhi* was identified as a PI3K that promotes bacterial entry and subsequent escape from phagosomes/autophagosomes to establish a replicative niche (36).

A previous study demonstrated that the PI3K inhibitor wortmannin blocks host cell invasion by SFG rickettsiae (24). However, little is known about the bacterial factor involved in PI metabolism modulation in host cells by SFG rickettsiae. In this study, we provide evidence to show that *R. rickettsii* (Sheila Smith strain, hereinafter referred to as *R. rickettsii*) utilizes a T4SS effector with class III PI3K activity to induce PI3P in the cytoplasm. Meanwhile, this effector facilitates autophagy influx by interacting with Beclin 1 (a.k.a. ATG6). Notably, the suppression of PI3P levels by overexpressing the phosphatidylinositol 3-phosphatase Myotubularin significantly impaired intracellular bacterial replication, highlighting the critical role of PI3P in *R. rickettsii* virulence.

## RESULTS

### Identification of a potential PI kinase from *R. rickettsii*

Modulation of PI metabolism is a well-established strategy employed by bacterial pathogens to facilitate host colonization (37). Among these, the effector Risk1 from *R. typhi* with both class І and class III PI 3-kinase activity functions to promote bacterial entry into host cells (36). Similar to other Rickettsia effectors, homologs of Risk1 are found across various Rickettsiales species such as *A1G_01070* in *R. rickettsii*, which shares 85% similarity with Risk1 (*RT0135*) (10).

We set out to identify *R. rickettsii* additional effectors, potentially participating in interfering with host PI metabolism. To this end, we analyzed proteins of this pathogen with the HHpred algorithm (https://toolkit.tuebingen.mpg.de/tools/hhpred) and identified the protein encoded by *A1G_04485* as a candidate effector with similarity to known PI kinases, including LepB_NTD (ranking first, identities: 16%, similarity: 17.5%) (38) and MavQ (ranking second, identities: 17%, similarity: 13.3%) from *Legionella pneumophila* (39, 40) and CtkA (cell translocating kinase A) (ranking third, identities: 18%, similarity: 15.8%) from *Helicobacter pylori* (41).

**Fig 1:**
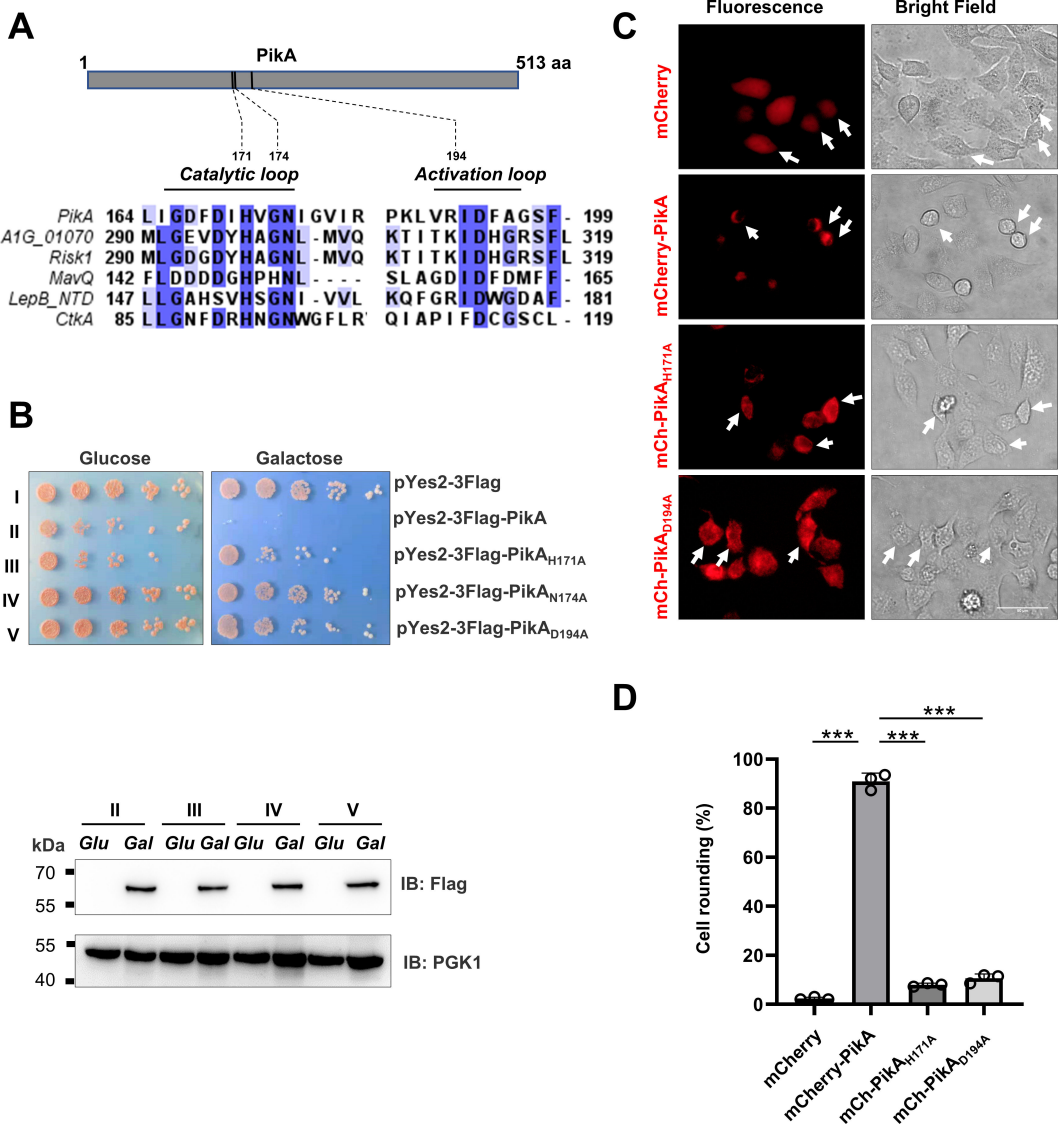
The predicted kinase motif in PikA is critical for its toxicity to eukaryotic cells. (**A**) Alignment of the sequences of the putative catalytic center in PikA with those of CTKA, LepB_NTD, MavQ, Risk1, and *A1G_01070* using the MUSCLE method. (**B**) The predicted His-Asn­Asp motif is essential for yeast toxicity of PikA. Cells of yeast strains expressing PikA or the indicated mutants from the galactose­inducible promotor were serially diluted and spotted on the indicated media. The plates were incubated at 30°C for 3 days before image acquisition. The expression of PikA and its derivatives was detected by immunoblotting with the anti-Flag antibody. The metabolic enzyme phosphoglycerate kinase (PGK1) was detected as a loading control. (**C**) Representative fluorescence and bright field images of HeLa cells transfected to express mCherry, mCherry-tagged PikA, and its enzymatically inactive mutants. Cells transfected for 18 h were used for image acquisition. Scale bars, 50 µm. (**D**) Quantitation of cell rounding in samples transfected to express PikA and its mutants. For each sample, at least 300 cells were scored and results (mean ± s.e.) shown were from three independent experiments. ***, *P* < 0.001.

Multiple sequence alignment of the protein coded by *A1G_04485* with related bacterial PI kinases using the MUSCLE method (42) revealed a potential kinase motif consisting of His_171_, Asn_174_, and Asp_194_ (Fig. 1; Fig. S1). In addition, *A1G_04485* shares 98.70% and 85.76% sequence similarity with *RC0797* from *R. conorii str. Malish 7* and *RT0527* from *R. typhi str. Wilmington*, respectively. Indeed, a phylogenetic tree of *A1G_04485* and its homologs constructed by three rounds of Position-Specific Iterated BLAST (PSI-BLAST) revealed that these proteins are distributed across *Rickettsia* spp., *Legionella* spp., and some unclassified species (Fig. S2). Considering that Rickettsial genomes have undergone significant reduction to adapt to their intracellular lifestyle (43), the widespread presence of *A1G_04485* homologs suggests its important role in the virulence of these bacteria. Due to its similarity to PI kinases, we designated *A1G_04485* as phosphatidylinositol kinase interfering with autophagy (PikA).

The expression of bacterial effectors involved in PI metabolism in yeast is a common method used to determine their impact on cellular processes (44). To investigate the role of the predicted active site in PikA on eukaryotic cells, we constructed yeast strains that expressed this effector and its mutants from the galactose-inducible promoter (45). The yeast strain harboring the plasmid expressing PikA was unable to grow on medium containing galactose. Mutations in His_171_, Asn_174_, or Asp_194_ abolished yeast toxicity without affecting protein stability (Fig. 1B), validating the importance of the predicted catalytic motif in its activity.

We next examined the effects of the putative PI kinase activity on mammalian cells. To this end, mCherry fusion of PikA, the His_171_ and Asp_194_ mutants were individually expressed in HeLa cells by transfection for 18 h prior to inspecting the morphology of the cells by fluorescence and bright-field imaging. We observed that cell-rounding occurred in about 90% cells expressing PikA, whereas less than 12% cells expressing the mutant proteins displayed this phenotype (Fig. 1C and D). To determine whether PikA affects the survival or morphology of mammalian cells, we employed the SYTOX Green nucleic acid stain, which revealed that ~50% of PikA-expressing HeLa cells were dead (Fig. S3A and B). Consistent with this result, PikA-expressing cells showed elevated LDH release, which represented ~20% cell death comparing to samples lysed by detergent. This effect required the predicted kinase activity as similarly expressed PikA_H171A_ or PikA_D194A_ mutants did not induce cell death (Fig. S3C). Thus, the predicted PI kinase activity of PikA interferes with one or more critical processes in eukaryotic cells.

### PikA is secreted into host cells through T4SS by a surrogate bacterium

**Fig 2:**
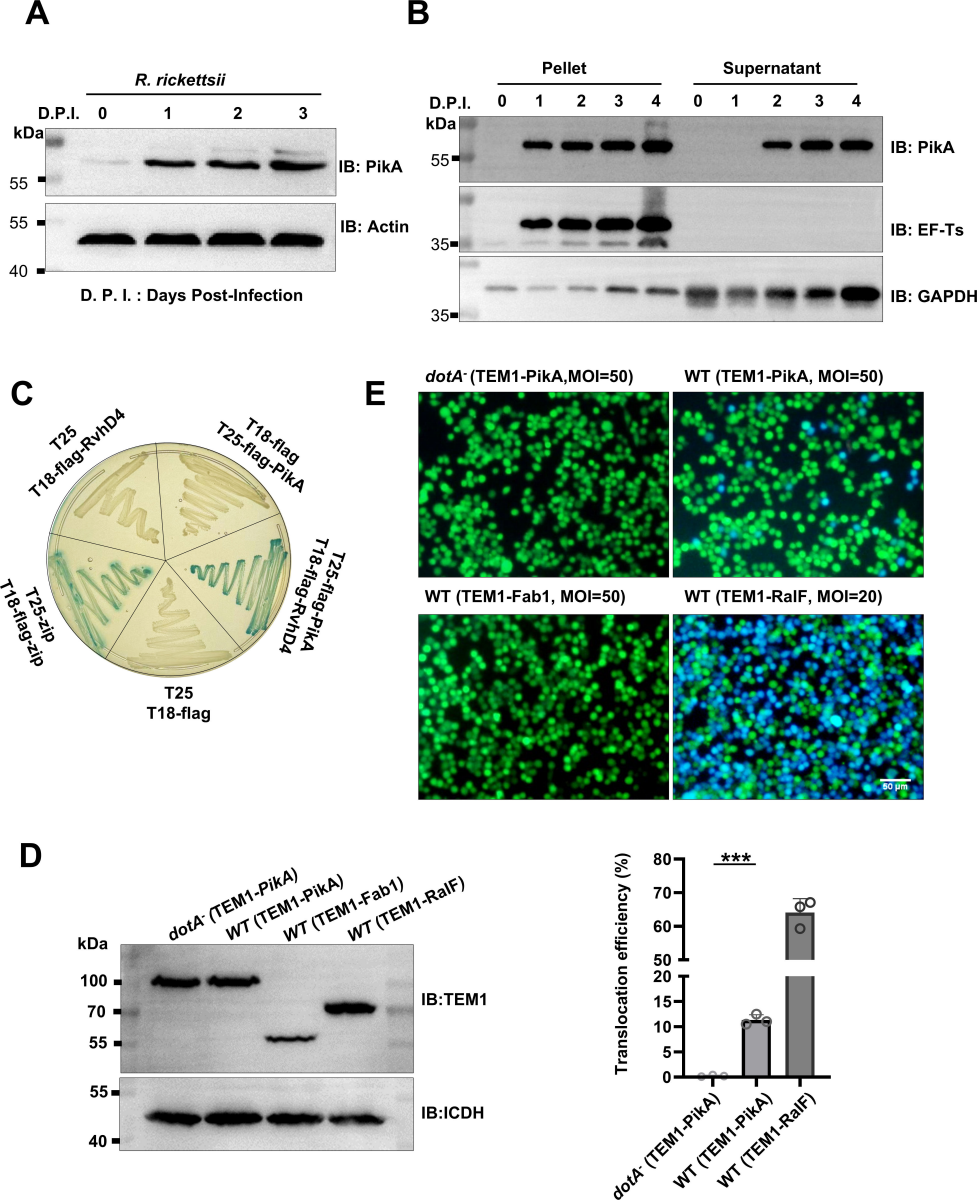
PikA is a substrate of Type IV secrete system. (**A**) HeLa cells were incubated with *R. rickettsii* (MOI = 1) for the indicated durations at 33°C. The presence of PikA in cell lysates was detected using antibodies specific for PikA, and actin was probed as a loading control. (**B**) HeLa cells infected with *R. rickettsii* (MOI = 1) for 0–4 days post-infection (D P.I.) were lysed with 0.1% Triton X-100, and lysates were fractionated by 10,000 *g* centrifugation. Uninfected cells were similarly processed as controls. Proteins in pellet and supernatant fractions were probed for PikA, the bacterial protein EF-Ts, and host protein GAPDH by immunoblotting. Similar results were obtained in three independent experiments. (**C**) Interactions between PikA and RvhD4 by a bacterial two-hybrid assay. The T25-PikA and T18-RvhD4 fusion were co-expressed in the reporter *E. coli* strain BTH101, strains harboring relevant combinations of plasmids were established as controls. Bacterial cells were steaked on LB plate containing X-Gal. Images were acquired after incubation at 37°C for 14 h. Note that strains capable of hydrolyzing X-Gal to form blue cells indicate positive interactions. (**D and E**) PikA is translocated into host cells by the *L. pneumophila* Dot/Icm transporter. Expression of the TEM1-PikA fusion. Cultures of *L. pneumophila* strains harboring the indicated plasmids were induced with 50 µM IPTG for 4 h at 37°C, and the fusion protein was probed by immunoblotting using anti-TEM antibodies. The metabolic enzyme isocitrate dehydrogenase (ICDH) was detected as a loading control. (**D**) Raw264.7 cells were challenged with Lp02 or Lp03 expressing the TEM1-PikA fusion at an MOI of 50 for 3 h prior to adding the CCF2-AM substrate. Samples incubated for an additional 2 h were used for image acquisition (E, top). Bar, 50 µm. Quantitation of cells emitting blue fluorescence signals. At least 300 cells were scored for each sample, and the results (mean ± s.e.) shown were from three independent experiments (E, bottom). ***, *P* < 0.001.

To determine whether PikA is expressed by *R. rickettsii* during infection, we generated polyclonal antibodies against PikA. Whereas PikA was barely detectable in samples from cells infected for less than 5 min, it became abundant 1 day post-infection, and the protein level was persistent for the entire experimental duration (Fig. 2A).

Next, we employed a fractionation experiment to evaluate the translocation of PikA into host cells during infection. HeLa cells infected with *R. rickettsii* for different durations were lysed with 0.1% Triton X-100, and the lysates were separated into supernatant and pellet fractions by high-speed centrifugation. The bacterial elongation factor Ts (EF-Ts) was probed in each fraction to assess bacterial integrity. The effectiveness of separation of the soluble cytoplasmic content from the pellet (including cell nuclei and *R. rickettsii* cells) was estimated by detecting the presence of the mammalian cytosolic glyceraldehyde-3-phosphate dehydrogenase (GAPDH). As expected, GAPDH was enriched in the supernatant, and EF-Ts was exclusively detected in the pellet of infected cells, indicating that the bacterial cells were intact after the samples had been processed by our lysis protocol. Importantly, PikA was detected in both the supernatant and pellet of infected HeLa cells from day 2 to day 4 samples (Fig. 2B). These results suggest that PikA is translocated into host cells by *R. rickettsii*. Notably, we observed that at day 1 although PikA was abundant in bacteria (pellet), it was undetectable in the soluble fraction (Fig. 2B), suggesting that PikA was translocated and exerts its function after the infection has been established.

Rickettsia species encode six distinct secretion systems, including two types of T4SS: P-T4SS and F-T4SS (46). Among these, P-T4SS has been identified as an evolutionarily conserved translocator referred to as Rickettsiales *vir* homolog (Rvh) T4SS (47), whereas F-T4SS only is present in some species of Rickettsia. Comparing to the well-characterized VirB/D4 T4SS of *Agrobacterium tumefaciens* (48), the Rvh system contains duplications of *virB9*, *virB8*, *virB4* but lacks a *virB5* homolog (49).

The Rvh T4SS of *R. rickettsii* has three ATPases: RvhD4, RvhB4, and RvhB11, which are situated at the base of the translocation apparatus (46). Among these, homologs of VirD4 serve as the coupling protein and are responsible for substrate recognition (50, 51). For example, VirD4 of *A. tumefaciens* promotes VirE translocation by directly interacting with its C terminal portion (52, 53). Specific interactions between substrates and RvhD4 have enabled the identification of T4SS effectors, including RaIF from *R. typhi* (33), RARP-2 from *R. rickettsii* (54), and Risk1 from *R. typhi* (36).

To determine whether PikA is transported by the Rvh T4SS, we first performed a bacterial two-hybrid assay to determine interactions between PikA and the coupling protein RvhD4 (55). Plasmids expressing T25-PikA and T18-RvhD4 fusions were transformed into the testing *E. coli* strain BTH101 (55, 56), which led to the expression of the reporter β-galactosidase (Fig. 2C). No expression of the reporter enzyme was detected in each of the control strains that expressed only one of the fusions (Fig. 2C). These results suggest that PikA interacts with RvhD4.

Since genetic manipulation techniques for *R. rickettsii* are currently unavailable, we utilized *L. pneumophila* as a surrogate to determine the T4SS-dependent translocation of PikA into host cells. *L. pneumophila* employs the Dot/Icm T4SS system to deliver effectors into host cells (57). In our study, RAW264.7 cells were infected with either the wild-type strain Lp02 (58) or the *dotA-*deficient strain Lp03 (59), both expressing the TEM1-PikA fusion, at a multiplicity of infection (MOI) of 50 for 2 h. Subsequently, the substrate CCF2/AM was added to the culture medium. Fluorescence imaging analysis revealed that, comparing to the Dot/Icm-deficient Lp03 strain, approximately 11% of RAW264.7 cells infected by Lp02 expressing the fusion exhibited a blue fluorescence signal upon excitation at 409 nm, indicating Dot/Icm-mediated translocation of the TEM1-PikA fusion into host cells. As a control, the TEM1-RaIF fusion (60) was translocated at a rate of about 60% at an MOI = 20, and no translocation was detected for the TEM1-FabI fusion (61) (Fig. 2D through E).

### PikA displays PI3-kinase activity toward PI and PI5P

**Fig 3:**
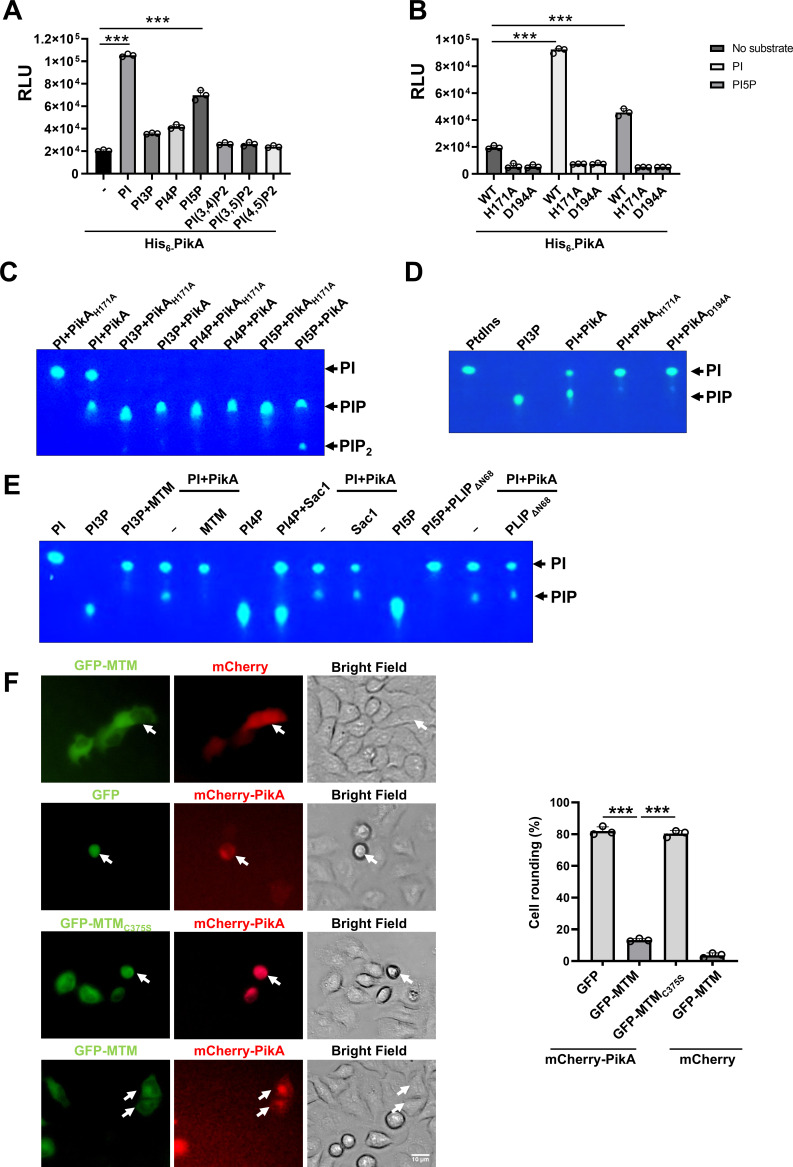
PikA phosphorylates PI to generate PI3P. (**A**) Biochemical assays for the kinase activity of PikA. Purified His_6_-PikA was incubated in reactions containing the indicated PI substrates, and the production of ADP was measured using the ADP-Glo Kinase Assay. RLU, relative luminescence units. (**B**) The His-Asn-Asp motif is critical for the kinase activity of PikA. His6-PikA or its mutants were incubated with PI or PI5P, and the activity was determined as described in A. (**C**) Determination of PikA-catalyzed production of a panel of PI substrates by TLC assays. His_6_-PikA or its inactive mutant His_6_-PikA_H171A_ was incubated with the indicated diC8-Bodipy-FL-PI species at 25°C for 14 h, and the products were separated by TLC. Images of the plates were acquired using the Goodsee-5 TLC imager. (**D**) Mutations in H171, D194 eliminate the monophosphorylation of PI catalyzed by PikA. Experiments were performed with the same procedure as described in panel C. (**E**) Dephosphorylation of PikA-produced products by PI phosphatases. The products produced by PikA were incubated with the indicated phosphatases at 37°C for 15 min, and the products were detected by TLC assays. (**F**) Suppression of PikA-induced cell rounding by MTM. HeLa cells were transfected to express the indicated protein combination for 14 h, and cell morphology was analyzed using a fluorescence microscope for image acquisition (left) and quantitation (right). Bar, 10 µm. Results (mean ± s.e.) shown were from three independent experiments, each done in triplicate. At least 300 cells were scored for each sample (right). ***, *P* < 0.001.

To determine its potential PI kinase activity, recombinant His_6_-PikA, His_6_-PikA_H171A_, and His_6_-PikA_D194A_ were purified from *E. coli* and analyzed using the ADP-Glo kinase assay (38, 39). The biochemical activity of these proteins was evaluated in the presence of a panel of PIs. In reactions containing His_6_-PikA, ATP, and each of the testing PIs, hydrolysis of ATP was detected when PI or phosphatidylinositol 5-phosphate (PI5P) was used as substrate, with PI exhibiting a more robust reactivity (Fig. 3A). In contrast, no ATP hydrolysis was observed in reactions receiving PI3P, PI4P, PI (3, 4)P_2_, PI (3, 5)P_2_, or PI (4, 5)P_2_ (Fig. 3A). In reactions containing PI or PI5P as substrate, neither His_6_-PikA_H171A_ nor His_6_-PikA_D194A_ induced ATP hydrolysis (Fig. 3B), indicating that the predicted kinase motif is essential for its PI metabolism activity.

We next employed a panel of Bodipy-FL labeled PIs to unveil the products generated by PikA by thin-layer chromatography (TLC) analysis which allows separation of PI species according to their polarity (62). Consistent with the results from the ADP Glo kinase assays, PI and PI5P received a phosphate group from ATP in reactions containing PikA but not the PikA_H171A_ mutant (Fig. 3C). A PI species migrating similar to PI3P was detected in reactions containing ATP, PI, and His_6_-PikA but was absent in reactions receiving PikA_H171A_ and PikA_D194A_ mutants (Fig. 3D). These results suggest that PikA modifies PI by mono-phosphorylation.

To pinpoint the exact PI species produced by PikA, we took advantage of several enzymes known to specifically hydrolyze phosphate groups at distinct positions of the inositol ring. Earlier studies have established that Myotubularin (63), Sac1p (64), and PLIP (65) specifically hydrolyze the phosphate groups at D3, D4, and D5 positions of the inositol ring in phosphoinositides, respectively. Incubation of PikA-generated product with the PI3P-specific phosphatase Myotubularin resulted in the formation of PI (Fig. 3E), indicating that PikA phosphorylates PI at the D3 position. Consistent with this notion, the product of PikA cannot be converted into PI either by the PI4P-specific phosphatase Sac1p (66) or the PI5P-specific phosphatase PLIP (65) (Fig. 3E). Furthermore, ectopic expression of Myotubularin but not its enzymatically inactive MTM_C375S_ in HeLa cells effectively suppressed the cell rounding phenotype induced by PikA. Of note, MTM itself did not detectably affect cell morphology (Fig. 3F). Taken together, these results indicate that PikA is a PI-3 kinase that catalyzes phosphorylation of the D3 position of PI and potentially of PI5P.

### Overexpression of PikA disrupts PI3P distribution

**Fig 4:**
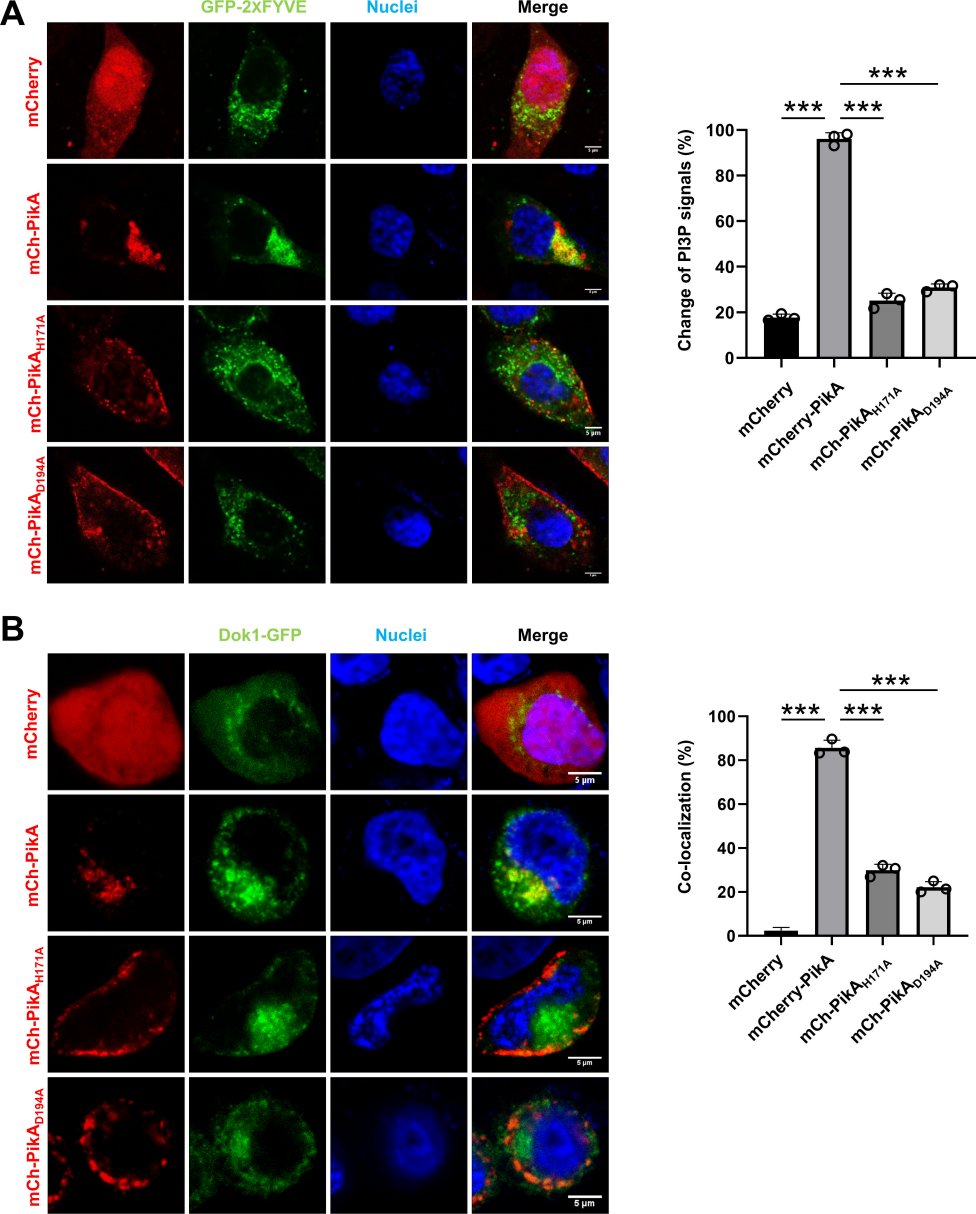
PikA disrupts the distribution of PI3P in cells and colocalizes with PI5P. (**A**) Distribution of the PI3P probe GFP-2xFYVE_Hrs_ in cells expressing and mCherry-PikA, mCherry-PikA_H171A_, or mCherry-PikA_D194A_. HeLa cells transfected to express the probe and mCherry-PikA or its mutants for 14 h were used to acquire images. Nuclei were stained with Hoechst (left). Bar, 5 µm. Quantitation of cells with an altered distribution of the PI3P probe. Results (mean ± s.e.) shown were from three independent experiments done in triplicate, at least 100 cells were counted for each sample (right). (**B**) Distribution of the PI5P probe Dok1-GFP in cells expressing mCherry-tagged PikA or its enzymatically inactive mutants. Samples were prepared as described in A (left). Bar, 5 µm. Quantitation of the co-localization between the PI5P probe and PikA or its mutants. Results (mean ± s.e.) shown were from three independent experiments done in triplicate, at least 100 cells were counted for each sample (right). ***, *P* < 0.001.

To further determine how PikA influences the intracellular PI3P level and its distribution, we employed the PI3P-binding domain FYVE from the hepatocyte growth factor-regulated tyrosine kinase substrate, fused to GFP (GFP-2 × FYVE) (67, 68). This probe was co-expressed with the mCherry-PikA chimera in Hela cells, or with mCherry fusions with the enzymatically inactive PikA_H171A_ and PikA_D194A_ mutants. In cells expressing PikA, the punctate fluorescence pattern of GFP indicative of PI3P became an aggregated pattern, and such changes did not occur in cells expressing the PikA_H171A_ and PikA_D194A_ mutants (Fig. 4A left). The change in PI3P distribution pattern was detected in approximately 90% of cells expressing PikA. In contrast, such changes were observed only in about 20% of cells expressing mutants (Fig. 4A right). As expected, the expression of MTM but not its catalytically inactive mutant MTM_C375S_ effectively rescued PikA-induced aggregation of PI3P signals, reverting them to a physiological punctate distribution pattern (Fig. S4).

To further analyze the impact of PikA on cellular PI5P, we employed the T cell-specific probe Dok1 that harbors an N-terminal PI5P binding PH motif (69) as a probe. We co-expressed the Dok1-GFP fusion with mCherry fused to PikA or its mutants in HeLa cells. Fluorescence imaging revealed that Dok1 colocalized with PikA but not with its mutants (Fig. 4B). Yet, fluorescence signals of cells expressing either PikA or the mutants did not display any difference in PI5P distribution (Fig. 4B), likely due to the relatively weak catalytic activity of PikA toward PI5P (Fig. 3A and C). As an additional control, we employed a GFP fusion of the PH domain of FAPP1, which specifically binds PI4P (70, 71), to further determine the impact of PikA. Co-expressed FAPP1-GFP fusion with mCherry-PikA, mCherry-PikA_H174A_, or mCherry-PikA_D194A_ did not cause a discernable change in the distribution of fluorescence signals (Fig. S5), further supporting the conclusion that PikA specifically catalyzes the production of PI3P.

### The PI3K activity of PikA is insensitive to Wortmannin

**Fig 5:**
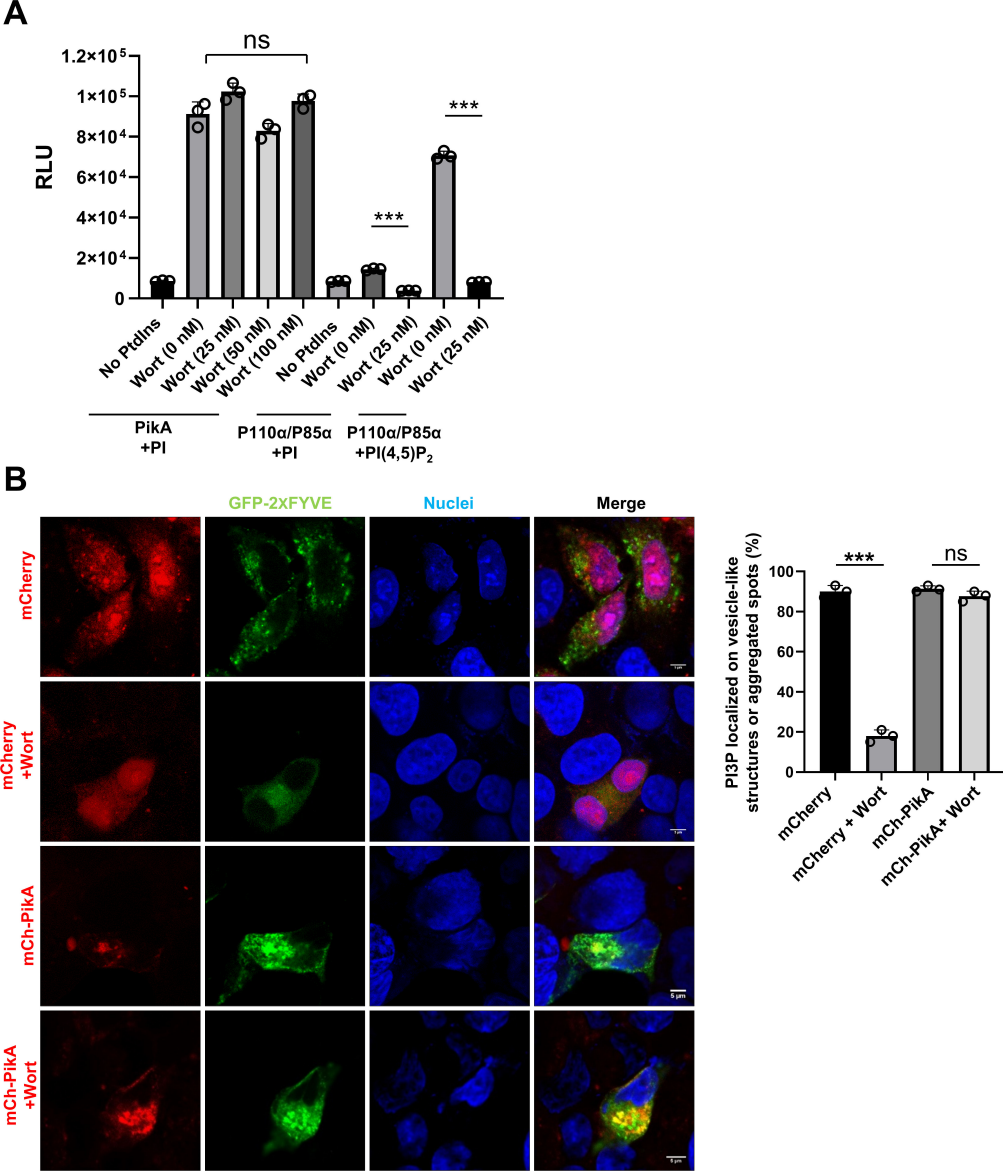
The PI3K activity of PikA is insensitive to wortmannin. (**A**) The PI3K inhibitor wortmannin does not detectably inhibit the activity of PikA. The inhibitor was added to PikA-catalyzed reactions for PI3P production at the indicated concentrations. Reactions using P110α/P85α with PI or PI (4, 5)P_2_ were established as controls. The activity was monitored by measuring ADP using the ADP-Glo assay. Results (mean ± s.e.) shown were from three independent experiments done in triplicate. (**B**) Wortmannin does not impact the distribution of GFP-2xFYVE_Hrs_ induced by PikA. HeLa cells transfected to express the indicated proteins for 14 h were subjected to image acquisition and scoring after adding 1 µM Wortmannin for 2 h. Control samples received DMSO (left). Bar, 5 µm. Quantitation of cells in which the PI3P probe was localized vesicle-like structures or aggregated spots. Results (mean ± s.e.) shown were from three independent experiments done in triplicate, at least 100 cells were scored for each sample (right). ns, not significant; ***, *P* < 0.001.

PI3Ks of eukaryotic origin often are sensitive to the inhibitor Wortmannin (Wort) (72), so is Risk1, the recently described PI3K from *R. typhi* (36). We, thus, used biochemical assays to determine whether PikA is sensitive to this inhibitor. A series of reactions were established with PI, PikA, and different concentrations of Wort, and the production of ADP was measured by the ADP-Glo kinase assay. No significant difference was observed among the reactions receiving different concentrations of Wort (Fig. 5A), indicating that the kinase activity of PikA was not affected by this inhibitor. As a control, we assessed the sensitivity of the PI3K p110α/p85 complex to Wort. Inclusion of 25 nM Wort in reactions containing PI (4, 5)P_2_ as a substrate effectively blocked the production of PI (3–5)P_3_ (Fig. 5A), confirming the inhibitory efficacy of Wort under our assay conditions. Additionally, we also examined the impact of Wort on the redistribution PI3P induced by PikA. Treatment with 1 µM Wort for 2 h caused the PI3P probe GFP-2xFYVE to diffuse from its normal punctuate pattern. Expressed PikA allowed the probe to maintain the original distribution pattern even in cells treated with Wort, further indicating that the activity of PikA is not affected by this inhibitor (Fig. 5B).

### PikA disturbs autophagy influx by acting on Beclin 1

**Fig 6:**
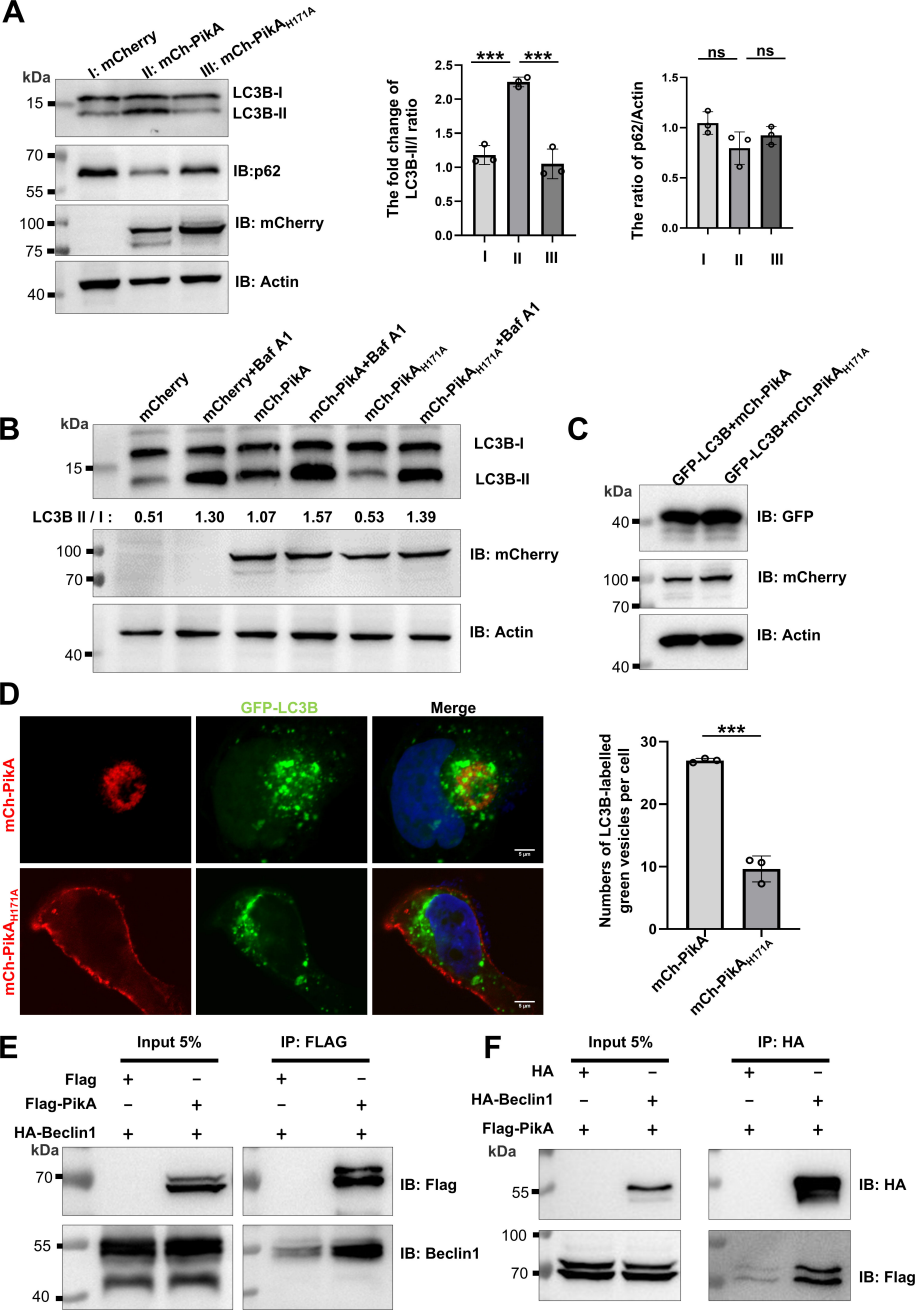
PikA disturbs autophagy by interacting with Beclin 1. (**A**) PikA induced the production of LC3B-II. Lysates of HEK293T cells transfected to express mCherry, mCherry-PikA, or mCherry-PikA_H171A_ for 14 h were probed for LC3B and p62 by immunoblotting, the expression of the PikA and its mutant was also probed, and actin was used as a loading control (left). Densitometry of LC3B using Fiji software from three independent experiments. Band intensity quantitation was performed to calculate the fold change of LC3B-II/LC3B-I ratio (right). (**B**) The effects of inhibitors on autophagy induced by PikA. HEK293T transfected to express mCherry, mCherry-PikA, or mCherry-PikA_H171A_ for 14 h were treated with 100 nM Bafilomycin A1 for 4 h, and LC3B processing was probed by immunoblotting. Actin was detected as a loading control. Data shown were one representative from three independent experiments with similar results. (**C and D**) PikA induced a notable increase in autophagosome formation. HeLa cells transfected to express GFP-LC3B and mCherry-PikA or mCherry-PikA_H171A_ for 14 h (**C**) were analyzed using a fluorescence microscope for image acquisition (D, left) (bars, 5 µm) and quantitation of the number of autophagosomes labeled by GFP-LC3B. Nuclei were labeled by Hoechst staining. Results (mean ± s.e.) shown were from three independent experiments done in triplicate, at least 100 cells were scored for each sample (D, right). ns, not significant; **, *P* < 0.01; ***, *P* < 0.005; ****, *P* < 0.001. (**E and F**) Interaction between PikA and Beclin1. Lysates of HEK293T cells transfected to express Flag-PikA and HA-Beclin1 for 14 h were subjected to immunoprecipitation with antibody specific for Flag (**E**) or HA (**F**), and co-purification of the interacting protein was detected by immunoblotting with the respective antibodies. Images shown were one representative of three independent experiments with similar results.

PI3P generated by members of the class III PI3-kinases (e.g., PtdIns3KC3 and Vps34 in yeast) at the phagophore assembly sites (PAS) is essential for the initiation of autophagy (73). To determine whether PikA regulates autophagy signaling, we examined the abundance of LC3 and p62 (a.k.a. sequestosome-1 [SQSTM1]), two common autophagy markers in HEK293T cells transfected to express PikA. Expression of PikA robustly increased the ratio of phosphatidylethanolamine-conjugated LC3B (LC3B-II) to LC3B-I, which did not occur in cells receiving the empty vector or the plasmid carrying the enzymatically inactive mutant mCherry-PikA_H171A_. A decrease of p62 was also detected in cells expressing PikA, but the ratio of p62/actin exhibited no statistically significant difference comparing to cells expressing mCherry-PikA_H171A_ or mCherry (Fig. 6A). Wortmannin did not affect the elevated ratio of LC3B-II/I induced by PikA (Fig. S6A), further supporting the notion that PikA does not impact endogenous signaling regulated by Wortmannin-sensitive PI3-kinases. The dampened LC3B-II/I response to bafilomycin A1 treatment in samples expressing PikA (wild-type Δratio 0.5 vs mutant Δratio 0.86) indicates that PikA likely induces autophagic flux impairment through mechanisms akin to Bafilomycin A1 (Fig. 6B). To further determine the effect of PikA on autophagy, we expressed GFP-LC3 in HeLa cells and quantitated GFP-labeled autophagosomes and found that PikA but not its inactive mutant PikA_H171A_ caused a threefold increase in the number of autophagosomes (Fig. 6C and D).

In light of earlier observation that the formation of a multiprotein complex containing the PI3-kinase Vps34 and the autophagy protein Beclin 1 is important for autophagy initiation (74), we examined whether PikA functions by incorporating into such protein complex. Yeast two-hybrid assays revealed potential interactions between Beclin 1 and PikA (Fig. S6B), which was further confirmed by immunoprecipitation (IP) (Fig. 6E and F). Consistent with these findings, co-expression of differently labeled PikA and Beclin 1 extensively co-localized (Fig. S6C). Importantly, the enzymatically inactive mutant PikA_H171A_ exhibited markedly reduced ability to co-isolate Beclin 1 (Fig. S6C and D). Taken together, our results imply that PikA mimics endogenous PI3-kinase to promote autophagy initiation by interacting with Beclin 1, which may allow the kinase to elevate PI3P at the PAS to accelerate autophagosome formation.

### PI3P produced by PikA promotes *R. rickettsii* intracellular replication

**Fig 7:**
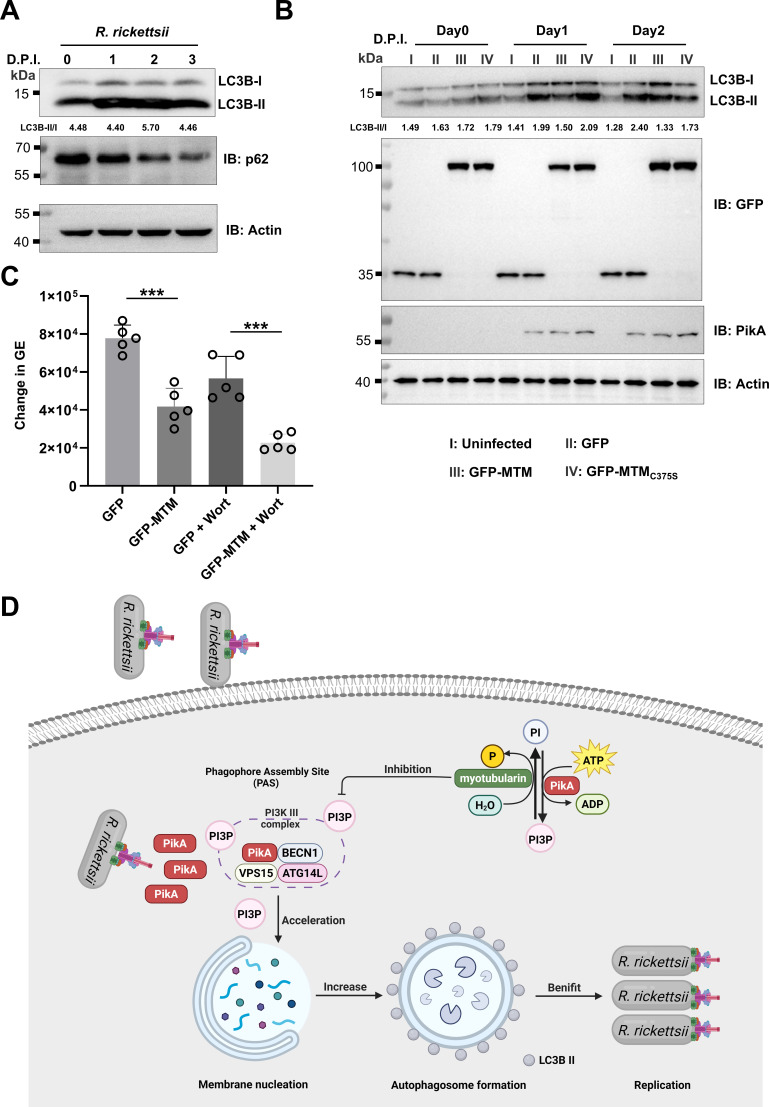
Inhibition of autophagy impairs *R. rickettsii* replication (**A**) *R. rickettsii* infection induced LC3B conjugation. HeLa cells infected with *R. rickettsii* at MOI = 1 were probed for LC3B and p62 by immunoblotting. Actin was probed as a loading control. (**B**) MTM counteracts the elevated level of autophagy induced by *R. rickettsii* infection. HeLa cells transfected to express GFP, GFP-MTM, or GFP-MTM_C375S_ for 14 h priority to infected with *R. rickettsii* at MOI = 1. The cell lysates at indicated timepoints of infection were probed for LC3B, PikA, and GFP. Actin was detected as a loading control. (**C**) Overexpression of MTM impairs intracellular bacterial growth. HeLa cells transfected to express GFP or GFP-MTM for 14 h were infected with *R. rickettsii* and treated with 1 µM wortmannin or DMSO (solvent control). Genome equivalent was determined 48 h after bacterial uptake. GE, genome equivalents/mL. Data (mean ± s.e.) shown one representative from three independent experiments each done in triplicate. (**D**) A model for autophagy modulation by PikA in *R. rickettsii* replication. PikA translocated into the host cell by *R. rickettsii* converts PI to PI3P, the effector also interacts with the host autophagosome initiation complex (Atg14L-Beclin 1-Vps15) to promote the formation of autophagosome. The PI3P phosphatase MTM reduced cellular PI3P, thus blocking *R. rickettsii* replication. ***, *P* < 0.001.

To assess the role of PikA in bacterial virulence, we first evaluated the level of p62 and LC3B species in cells infected with *R. rickettsii*, which revealed that *R. rickettsii* infection induced autophagy, evidenced by an increase of LC3B-II and down-regulation of p62 (Fig. 7A). To determine the effect of PI3P on autophagy in cells infected by *R. rickettsii*, we analyzed the abundancy of LC3B-I and LC3B-II in infected cells. The increase in LC3B-II caused by *R. rickettsii* infection was suppressed by overexpressing Myotubularin (Fig. 7B). Finally, we determined the effect of Myotubularin on bacterial replication and found that ectopic expression of this PI3P phosphatase inhibited intracellular bacterial replication (Fig. 7C). Thus, PikA induces the production of PI3P to interfere with autophagy influx, promoting *R. rickettsii* virulence.

## DISCUSSION

Modulation of PI metabolism is a common strategy used by bacterial pathogens (37). These strategies often involve the deployment of bacterial effectors of PI kinase or phosphatase activity to override the finely tuned PI metabolism in host cells. For example, *Francisella tularensis* uses the PI3K OpiA to generate PI3P on the phagosome to delay its maturation (44). *L. pneumophila* employs the MavQ-LepB-SidF axis to enrich PI4P on its phagosome (38, 39, 75), where it serves as the anchor for a subset of *Dot/Icm* effectors and for mimicking the property of the *cis*-Golgi compartment to facilitate the fusion of vesicles originating from the ER (76, 77). We report here that *R. rickettsii* utilizes the PI3K activity of PikA to promote bacterial virulence by enhancing autophagy. Similar to MavQ, a PI3K from *L. pneumophila* (39), PikA disrupted the distribution of PI3P in cells and this disruption cannot be blocked by the PI3K inhibitor wortmannin (78). This feature is shared by most PI kinases of bacterial origin probably due to divergence in structures and mechanism of catalysis (37). An exception in this regard is Risk1, a PI3K from *R. typhi* with both class I and class III activities that facilitates bacterial invasion and growth by subverting phosphoinositide metabolism (36).

PikA interacts with Beclin 1, and the interaction is promoted by its kinase activity, suggesting that PI3P facilitates the binding between these two proteins. This phenomenon is similar to the interaction between the mammalian PI3K Vps34, which also interacts with Beclin 1 in a manner that is impacted by its kinase activity: Cdk1-mediated phosphorylation of Vps34 downregulates its kinase activity and negatively impacts its binding to Beclin 1 (79). Despite these interesting observations, the mechanism underlying how PI3P enhances such protein interaction is unknown.

Autophagy is a branch of innate immunity against intracellular invaders, and successful pathogens have evolved various strategies to counteract autophagy-mediated killing or to co-opt autophagy for their benefits (80, 81). For example, *Listeria monocytogene*s and *Rickettsia parkeri* utilize surface protein ActA and OmpB to block autophagic recognition, respectively (82, 83). *L. pneumophila* uses the protease RavZ to irreversibly deconjugate LC3B II from membrane phosphatidylethanolamine (84), whereas SopF of *Salmonella Typhimurium* inhibits the V-ATPase-ATG16L autophagy axis by catalyzing ADP-ribosylation on Gln124 of ATP6V0C (85). Differing from these scenarios, PikA appears to promote autophagy to benefit intracellular growth of *R. rickettsii*. Co-option of autophagy to benefit bacterial pathogens is not unprecedented. *Coxiella burnetii* employs effectors CvpB and CvpF to convert its phagosome called Coxiella-containing vacuole (CCV) with features of autophagolysosome (86, 87). CvpB recruits host PI3P to the surface of the CCV while simultaneously inhibiting the activity of the PI5-kinase PIKfyve. This dual mechanism leads to PI3P accumulation on the surface of CCV, which facilitates the recruitment of the autophagosomal machinery to CCVs for optimal homotypic fusion of the Coxiella-containing compartments (86). CvpF functions by activating Rab26 leading to recruitment of the LC3B to CCV induces autophagy to promote bacterial multiplication (87). Although its biochemical activity is unknown, Ast1 of *Anaplasma phagocytophilum* interacts with Beclin 1 on the bacterial phagosome to promote autophagy initiation (88). Finally, Etf-1 of *Ehrlichia chaffeensis* facilitates bacterial growth by binding to Rab5 and hijacking the autophagy initiation PI3K complex (89). In agreement with these results, in mice deficient in *ATG5* or *ATG16L1*, bacterial load of *R. australis* is significantly lower than in wild-type mice (90, 91), suggesting that autophagy promotes to its virulence. Autophagy may facilitate nutrient delivery to the pathogen, as exemplified by Etf-1 of *Ehrlichia chaffeensis* (89). In addition, autophagy can counteract the production of pro-inflammatory cytokines such as IL-1β and IL-1α by infected macrophages (90–92). Whether and how other aspects of autophagy contribute to intracellular bacterial growth require further study.

*Rickettsia* spp. employ two major classes of virulence factors: outer membrane proteins (OMPs) and T4SS effectors. Transposon mutagenesis has identified several OMPs such as OmpB (critical for autophagy evasion (82, 93–95)), Sca2 (required for cell-to-cell spread [31]), Sca4 (mediates intercellular tension [96]), and Pat1 (important for replication in macrophages [97]) critical for optimal virulence. Site-directed knockout mutagenesis has confirmed phospholipase D as a key virulence factor (98). Three T4SS effectors, RalF, PARP2, and Risk1, have been described although their roles in virulence have not yet been evaluated by mutants lacking these genes. The discovery of PikA establishes a link among PI3P, autophagy, and *R. rickettsii* virulence. Interference of cellular PI3P levels impairs intracellular replication of *R. rickettsii* (Fig. 7D), suggesting that this effector plays an important role in virulence. Future work aiming at identifying inhibitory compounds targeting the PI3K activity of PikA will offer both a means to clarify its role in *R. rickettsii* virulence and a potential avenue for therapeutic intervention against infections caused by this pathogen.

## MATERIALS AND METHODS

### Cell culture and bacterial infections

Vero, HeLa, RAW 264.7, and HEK293T cells purchased from the American Type Culture Collection (ATCC) were cultured in Dulbecco’s modified Eagle’s medium (DMEM) supplemented with 10% FBS, 1% HEPEs, and 1% penicillin-streptomycin at 37°C in a 5% CO2 incubator.

*R. rickettsii* (Sheila Smith) was cultured in Vero cells and isolated by isopycnic density gradient centrifugation in a BSL-3 laboratory, as previously described (99, 100). Briefly, confluent monolayers of Vero cells grown in DMEM supplemented with 2% FBS and 2 mM L-glutamine were infected with *R. rickettsii* at MOI = 1 and then incubated at 33°C with 5% CO_2_ until 50% of the monolayer was disrupted due to bacterial replication. The number of *R. rickettsii* cells and viable rickettsial bacteria in suspension was detected by quantitative polymerase chain reaction (qPCR) (101) and plaque assay (102), respectively.

### *R. rickettsii* purification

The culture supernatant of Vero cells infected with *R. rickettsii* was replaced with PBS in culture bottle at 5 day post-infection. Cells were then scraped and transferred into 50 mL centrifuge tubes and placed on ice. Cell disruption was performed using a UP-250 ultrasonic cell mill probe (XINZHI, Ningbo, China) set to 30% intensity, with an oscillation cycle of 2 s followed by a 2 s pause, repeated for a total duration of 1 min. The samples were centrifuged at 1,680 *g* for 10 min at 4°C. The supernatant was subjected to high-speed centrifugation at 12,000 *g* for 10 min at 4°C. The resulting pellet was then resuspended with SPG buffer and stored at −80°C.

### Secretion assay

HeLa cells, either uninfected or infected with *R. rickettsii* for different durations, were lysed on ice for 0.5  h in PBS containing 0.1% Triton X-100 and protease phosphatase inhibitors. After centrifugation at 10,000 * g* for 10  min, the pellet containing insoluble components and intact bacteria was collected. The supernatant, which contained host cytosolic proteins and rickettsial secreted effectors, was concentrated by precipitation using trichloroacetic acid and sodium deoxycholate (36). Precipitated proteins were pelleted by centrifugation at 16,000 * g* for 10  min and washed with cold acetone. Both pellet and supernatant samples were immunoblotted with antibodies against PikA, EF-Ts, and GAPDH.

### DNA manipulation and plasmid construction

We amplified the coding region of *pikA* from the genomic DNA of *R. rickettsii*. The H171A, N174A, and D194A mutants of *pikA* were generated by site-directed mutagenesis with primers containing the desired mutations. To assess the toxicity of PikA and its mutants to yeast, we inserted the gene into pYES2/CT which drives gene expression by the *Gal1* promoter (Invitrogen).

To express proteins in mammalian cells, the coding region of *pikA* and its derivatives was inserted into pmCherry-C1 (Clontech), pCMV4×Flag (103), and pAPH-HA (104). The PI3P probe GFP-2xFYVE_Hrs_ and PI4P probe PH_FAPP1_ have been described elsewhere (38). The gene encoding the PI5P-binding Dok1 was inserted into pEGFP-N1 to serve as an indicator for PI5P (69). For expression and purification of PikA and its mutants, we cloned the coding region of the alleles into pET28a (Novagen). The cDNA of *myotubularin 1*, *Sac1p,* and *PLIP* was synthesized by GenScript and cloned into pETSumo (Thermo Fisher). The bacterial strains, plasmids, and primers used in this study were listed in Table S1.

### Protein purification

To produce His_6_-tagged proteins, pET28a carrying *pikA* or its mutants was transformed into *Escherichia coli* BL21 (DE3) (TransGen), and the resulting bacterial strains were cultured in LB medium with 30 µg/mL kanamycin to a density at OD600 = 0.6–0.8. Induction was carried out with 200 µM IPTG at 18°C for 16–18 h. Cells were harvested by centrifugation at 12,000 *g*, lysed using a cell homogenizer (JN-mini, JNBIO, Guangzhou, China), and the soluble lysates were harvested by spinning at 12,000 *g* for 20 min at 4°C. His_6_-tagged proteins were purified using Ni^2+^-NTA beads (QIAGEN). In each case, beads bound to the target protein were washed with threefold volumes of lysis buffer containing 20 mM imidazole. Proteins were eluted with 250 mM imidazole in PBS and dialyzed overnight in buffer containing 150 mM NaCl, 20 mM Tris-HCl (pH 7.5), and 10% glycerol.

### Bacterial 2-hybrid assays

Flag-tagged *PikA* and *RvhD4* were cloned into the plasmids of pKT25 and pUT18C (105), respectively. Different combinations of plasmids, as described in the article, were transformed into *Escherichia coli* BTH101 (56) and streaked on the LB agar plate supplemented with 80 µg/mL X-Gal, 30 µg/mL kanamycin, and 100 µg/mL ampicillin. If protein-protein interaction occurrs, cAMP synthesis leading to the activation of the *lacZ* gene, subsequently encodes β-galactosidase which effectively hydrolyzes chromogenic substrate X-Gal and forms blue colonies (55, 106). pKT25-Zip interacted with pUT18-Zip depending on the leucine zipper motif (105), which was used as a positive control (106).

### Protein translocation assay using β-lactamase as a reporter

*PikA* was cloned into pZLQ-Flag-TEM1 (107) to express the TEM1-PikA fusion. The plasmid was electroporated into wild-type or a *dotA*^−^ deficient *L. pneumophila* (57), respectively, and the resulting bacterial strains were used to infect RAW264.7 cells to assess Dot/Icm-mediated protein translocation. After 4 h of induction with 50 µM IPTG, *L. pneumophila* expressing the TEM1-PikA fusion was used to infect Raw264.7 cells at MOI of 50. After 3 h infection, 6 × CCF2AM substrate loading solution (ThermoFisher, Cat# K1023) was added to the culture medium at final concentration of 1× and incubated at room temperature for 2 h in the dark. Images were acquired using an IX-83 fluorescence microscope. If the protein of interest was transferred by T4SS, the fused β-lactamase cleaves CCF2 which is excited by 409 nm laser, producing a blue fluorescence signal (450 nm). If not, intact CCF2 emits a green fluorescence signal (520 nm) (108). The ratio of blue cells was determined by enumerating cells (*n* = 300) from three randomly selected images. The expression of TEM1 fusions in *L. pneumophila* was detected by immunoblotting.

### Antibodies and immunoblotting

Rabbit polyclonal antibodies were produced by immunized New Zealand white rabbits with purified His_6_-PikA and His_6_-EF-Ts (elongation factor thermos stable) following a standard protocol (Jiaxuan Biotechnology Co., Ltd., Beijing, China).

For immunoblotting, NP-40 lysis buffer (Beyotime, Cat# P0013F) additional adding protease inhibitor cocktail (Roche, Cat# 11697498001) was used to lyse transfected or infected cells. Lysates were prepared by adding 5 × SDS loading buffer and heated at 100°C for 10 min.

For immunoprecipitation, lysates were incubated with Flag or HA affinity beads on a rotatory at 4°C for overnight. Pellet the beads by centrifugation and carefully remove the supernatant to discard unbound proteins. Wash beads 3–5 times with cold lysis buffer to remove nonspecific binding. Resuspend beads in 30 µL 1 × SDS loading buffer and heat at 100°C for 10 min.

Total proteins were separated by SDS-PAGE and then transferred onto hydrophobic PVDF membranes (Merck, Cat# ISEQ00010) for following blocked with 5% nonfat milk. Proteins were detected by incubation with appropriate primary antibodies at indicated dilutions: anti-TEM (Abcam, Cat# ab12251, 1:3,000), anti-ICDH (1:10,000) (109), anti-PikA (1:1,000), anti-mCherry (Proteintech, Cat# 26765-1-AP, 1:2,000), anti-GFP (Proteintech, Cat# 66002-1-Ig, 1:2,000), anti-PGK (Abcam, Cat# ab113687, 1:2,500), anti-β-actin (Proteintech, Cat# 66009-1-Ig, 1:10,000), anti-Flag (Sigma, Cat# F1804, 1:3,000), anti-HA (Sigma, Cat# H3663, 1:3,000), anti-LC3B (Abcam, Cat# ab192890, 1:1,000), anti-P62/SQSTM1(Proteintech, Cat# 18420-1-AP, 1:1,000) and anti-Beclin1 (Proteintech, Cat#11306-1-AP, 1:1,000). After incubation on a rotatory at 4°C for overnight, PVDF membranes were washed three times in TBS-T, incubated with appropriate secondary antibodies conjugated to HRP for 1 h at room temperature, and then visualized by Tanon 5200 Chemiluminescent Imaging System.

### Yeast manipulation and toxicity assays

Plasmids were transformed into *Saccharomyces cerevisiae* strain W303 (110) by the lithium acetate (LiAc) method (111) and plated on SD (synthetic dropout) Ura^−^ medium containing 2% glucose. Plates were incubated at 30°C for a minimum of 3 days before use in toxicity assays.

Toxicity was evaluated as follows: testing strains were grown for 14 h at 30°C in 5 mL SD Ura^−^ medium containing 2% glucose. Cultures were fivefold serially diluted in sterile water, and 10 µL of each dilution was spotted onto SD Ura^−^ plates containing 2% glucose or galactose. Plates were incubated at 30°C for 3 days before image acquisition.

To detect protein expression, cells of the testing strains were grown in medium containing 2% glucose to a cell density of OD600 = 0.4. The cells were then suspended in medium containing 2% galactose, and the induction was allowed to proceed for 6 h. Cell lysates were prepared following an established protocol (112).

### Transfection and immunofluorescence assay

To identify whether PikA and its mutants ectopically expressed in HeLa cells disrupts the distribution of phosphoinositides, we cloned PikA and its derivatives into pmCherry-C1 (Clontech) and a set of phosphoinositides probes are fused into ORF of eGFP (Table S1). HeLa cells were cultured on the coverslips in 24-well plates prior to transfection. Co-transfection with indicated plasmids was performed using Lipofectamine 3000 transfection reagent (Invitrogen) according to the manufacturer’s instructions. After incubation of 16–18 h, HeLa cells were observed by fluorescence microscope to ensure the expression of plasmids.

For fluorescence staining, HeLa cells were fixed with 4% paraformaldehyde for 10 min at room temperature (RT). After washed with PBS, the cells were permeabilized for 10 min in PBS containing 0.5% Triton X-100 and then stained nuclei with Hoechst dye (1:5,000) for 15 min. Finally, the coverslips were placed upside down on the cover glass and sealed with nail polish. Images were acquired by a Zeiss LSM 880/NLO laser scanning confocal microscope.

### Thin-layer chromatography

A series of Bodipy-FL labeled phosphoinositides (Echelon Biosciences) were used as substrates for the kinase assay. The kinase reaction and following performance were carried out according to published procedures (38, 39). Briefly, 200 ng purified PikA or its mutants were incubated with 10 µM substrates in the presence of 250 µM ATP in kinase buffer containing 40 mM Tris–HCl (pH 7.5), 20 mM MgCl_2_, 1 mM dithiothreitol (DTT), and 0.025 ng/mL of BSA at 25°C overnight. Reaction products were dried in a Speed-Vac for 30 min.

For reactions containing phosphatases, the products were resuspended in 20 µL of phosphatase buffer containing 50 mM ammonium carbonate (pH 8.0) and 2 mM DTT. Then, 200 ng recombinant phosphatase was added to the mixture and incubated at 37°C for 15 min. The dried pellets were resuspended in 10 µL buffer containing methanol/isopropanol/acetic acid (vol/vol/vol, 5/5/2) and spotted onto the TLC plate (silica gel 60 F254) which activated with methanol/water (vol/vol, 3/2) containing 1% potassium oxalate (75) and dried for 1 h at 65°C. The samples on the plate were developed and separated into a mobile phase consisting of chloroform/methanol/acetone/glacial acetic acid/water (vol/vol/vol/vol/vol, 70/50/20/20/20). After 1 h of development, the positions of fluorescent phosphoinositides were visualized by a Goodsee-5 TLC imager.

### ADP-glo kinase assay

A panel of diC8 PIs purchased from Echelon Biosciences was individually dissolved in sterilized deionized water to final stock solutions at 1 mM. The kinase activity was determined by the ADP-Glo Kinase Assay (Promega) in 96-well white polystyrene plates following the manufacturer’s protocol. Purified enzymes (200 ng) were incubated with 10 µM PI substrates and 250 µM ATP in 25 µL of kinase buffer for 60 min at 25°C in the dark. The reaction was terminated by adding 25 µL of ADP-Glo reagent and incubating for 40 min at 25°C. Subsequently, 50 µL of detection reagent was added, and the mixture was incubated for an additional 40 min at 25°C. Luminescence was measured using a Synergy H1 Hybrid Multi-Mode Microplate Reader (BioTek).

### Yeast two-hybrid assay

*PikA* gene from *R. rickettsii* was cloned into the yeast two hybrid vector pGBKT7 (Clontech) in fusion with the GAL4 DNA-binding domain, and *BECN1* gene from HeLa cells was cloned into pGADGHM (Clontech) in fusion with the GAL activation domain. The indicated plasmids in Fig. S6B were transformed into the yeast strain AH109 (MKBio) by the LiAc method, and the resulting strains were streaked onto the SD Trp^−^, Leu^−^ agar medium. After growth on the plate for 3–4 days at 30°C, single colonies from different groups were subjected to fivefold serial dilution and spotted onto the plate SD agar with quadruple amino acid dropout (SD Trp^−^/Leu^−^/His^−^/Ura^−^) and double amino acid dropout (SD Trp^−^/Leu^−^) medium. Images were acquired after incubation for another 3–4 days at 30°C.

### qPCR and WB of *R. rickettsii* infected HeLa cells

HeLa cells were seeded in a 12-well plate at a density of 1 × 10^5^ cells per well and then transfected with the recombinant plasmid using jetPRIME (Polyplus, France, Cat# 101000046) according to the manufacturer’s instructions. The transfected cells were infected with *R. rickettsii* at MOI = 1 for 3 h in a 5% CO2 incubator at 33°C. Following infection, cells were washed with PBS and cultured in fresh medium. Finally, cells were collected at different times post-infection, and DNA and protein samples were extracted from the collected cells for qPCR and immunoblotting analysis, respectively.

HeLa cells infected in individual wells of 12-well plates were washed by PBS and scraped with the AL buffer supplied by DNeasy Blood &Tissue kit (Qiagen, GmbH, Germany). Total DNA from the cell pellets was extracted according to the instruction of kit, and the DNA from each sample was eluted with 100 µL of elution buffer. The rickettsial DNA copies were measured using qPCR targeting a 76 bp fragment of *ompB* gene with primers and a TaqMan-MGB probe. Reactions were performed with Taqman Universal Master mix on an Applied Biosystems QuantStudio 3. Each qPCR run included a standard curve from 10-fold serial dilutions of a known concentration of plasmid DNA carrying *R. rickettsii ompB*.

### Statistical analyses

Quantitative data were processed and analyzed by GraphPad Prism 9 software (GraphPad Prism, San Diego, CA, USA). Student’s *t*-test was used to compare the mean levels between two groups, each with at least three independent samples.

## SUPPLEMENTAL MATERIAL

Figure S1 mbio.02284-25-s0001.TIF The H-N-D motif of PikA is conserved in many bacterial effectors with PI kinase activity.

Figure S2 mbio.02284-25-s0002.TIF Distribution of PikA homologs in different groups of *Rickettsia* and Legionella species.

Figure S3 mbio.02284-25-s0003.TIF Expression of PikA caused mammalian cell death.

Figure S4 mbio.02284-25-s0004.TIF MTM reverses PikA-triggered clustering of PI3P signals to vesicle-like distribution.

Figure S5 mbio.02284-25-s0005.TIF PikA does not influence the distribution of PI4P.

Figure S6 mbio.02284-25-s0006.TIF Kinase activity-dependent interaction between PikA and Beclin1.

Legends mbio.02284-25-s0007.docx Legends for Fig. S1-S6.

Table S1 mbio.02284-25-s0008.docx Bacterial strains, plasmids, and primers used in this study.

ASM does not own the copyrights to Supplemental Material that may be linked to, or accessed through, an article. The authors have granted ASM a non-exclusive, world-wide license to publish the Supplemental Material files. Please contact the corresponding author directly for reuse.

## References

[1] Walker DH, Ismail N. 2008. Emerging and re-emerging rickettsioses: endothelial cell infection and early disease events. Nat Rev Microbiol 6:375–386. doi:10.1038/nrmicro1866 18414502

[2] Radulovic S, Price PW, Beier MS, Gaywee J, Macaluso JA, Azad A. 2002. Rickettsia-macrophage interactions: host cell responses to Rickettsia akari and Rickettsia typhi. Infect Immun 70:2576–2582. doi:10.1128/IAI.70.5.2576-2582.2002 11953398 PMC127898

[3] Rumfield C, Hyseni I, McBride JW, Walker DH, Fang R. 2020. Activation of ASC inflammasome driven by toll-like receptor 4 contributes to host immunity against rickettsial infection. Infect Immun 88:e00886-19. doi:10.1128/IAI.00886-19 32014896 PMC7093143

[4] Azad AF, Beard CB. 1998. Rickettsial pathogens and their arthropod vectors. Emerg Infect Dis 4:179–186. doi:10.3201/eid0402.980205 9621188 PMC2640117

[5] Huang D, Luo J, OuYang X, Song L. 2022. Subversion of host cell signaling: the arsenal of Rickettsial species. Front Cell Infect Microbiol 12:995933. doi:10.3389/fcimb.2022.995933 36389139 PMC9659576

[6] Voss OH, Rahman MS. 2021. Rickettsia-host interaction: strategies of intracytosolic host colonization. Pathog Dis 79:ftab015. doi:10.1093/femspd/ftab015 33705517 PMC8023194

[7] Quintal D. 1996. Historical aspects of the rickettsioses. Clin Dermatol 14:237–242. doi:10.1016/0738-081x(96)00007-7 8727125

[8] Dantas-Torres F. 2007. Rocky mountain spotted fever. Lancet Infect Dis 7:724–732. doi:10.1016/S1473-3099(07)70261-X 17961858

[9] Parola P, Paddock CD, Raoult D. 2005. Tick-borne rickettsioses around the world: emerging diseases challenging old concepts. Clin Microbiol Rev 18:719–756. doi:10.1128/CMR.18.4.719-756.2005 16223955 PMC1265907

[10] McLeod MP, Qin X, Karpathy SE, Gioia J, Highlander SK, Fox GE, McNeill TZ, Jiang H, Muzny D, Jacob LS, Hawes AC, Sodergren E, et al.. 2004. Complete genome sequence of Rickettsia typhi and comparison with sequences of other rickettsiae. J Bacteriol 186:5842–5855. doi:10.1128/JB.186.17.5842-5855.2004 15317790 PMC516817

[11] Raoult D, Ndihokubwayo JB, Tissot-Dupont H, Roux V, Faugere B, Abegbinni R, Birtles RJ. 1998. Outbreak of epidemic typhus associated with trench fever in Burundi. Lancet 352:353–358. doi:10.1016/s0140-6736(97)12433-3 9717922

[12] Binder AM, Nichols Heitman K, Drexler NA. 2019. Diagnostic methods used to classify confirmed and probable cases of spotted fever rickettsioses - United States, 2010-2015. MMWR Morb Mortal Wkly Rep 68:243–246. doi:10.15585/mmwr.mm6810a3 30870409 PMC6421962

[13] Walker TS. 1984. Rickettsial interactions with human endothelial cells in vitro: adherence and entry. Infect Immun 44:205–210. doi:10.1128/iai.44.2.205-210.1984 6425214 PMC263501

[14] Blanc G, Ngwamidiba M, Ogata H, Fournier PE, Claverie JM, Raoult D. 2005. Molecular evolution of rickettsia surface antigens: evidence of positive selection. Mol Biol Evol 22:2073–2083. doi:10.1093/molbev/msi199 15972845

[15] Uchiyama T. 2003. Adherence to and invasion of Vero cells by recombinant Escherichia coli expressing the outer membrane protein rOmpB of Rickettsia japonica. Ann N Y Acad Sci 990:585–590. doi:10.1111/j.1749-6632.2003.tb07431.x 12860694

[16] Martinez JJ, Seveau S, Veiga E, Matsuyama S, Cossart P. 2005. Ku70, a component of DNA-dependent protein kinase, is a mammalian receptor for Rickettsia conorii. Cell 123:1013–1023. doi:10.1016/j.cell.2005.08.046 16360032

[17] Chan YGY, Cardwell MM, Hermanas TM, Uchiyama T, Martinez JJ. 2009. Rickettsial outer-membrane protein B (rOmpB) mediates bacterial invasion through Ku70 in an actin, c-Cbl, clathrin and caveolin 2-dependent manner. Cell Microbiol 11:629–644. doi:10.1111/j.1462-5822.2008.01279.x 19134120 PMC2773465

[18] Hillman RD, Baktash YM, Martinez JJ. 2013. OmpA-mediated rickettsial adherence to and invasion of human endothelial cells is dependent upon interaction with α2β1 integrin. Cell Microbiol 15:727–741. doi:10.1111/cmi.12068 23145974 PMC3610814

[19] Noriea NF, Clark TR, Hackstadt T. 2015. Targeted knockout of the Rickettsia rickettsii OmpA surface antigen does not diminish virulence in a mammalian model system. mBio 6:mBio doi:10.1128/mBio.00323-15 PMC4453529 25827414

[20] Noriea NF, Clark TR, Mead D, Hackstadt T. 2017. Proteolytic cleavage of the immunodominant outer membrane protein rOmpA in Rickettsia rickettsii. J Bacteriol 199:1–13. doi:10.1128/JB.00826-16 PMC5331673 28031280

[21] Riley SP, Goh KC, Hermanas TM, Cardwell MM, Chan YGY, Martinez JJ. 2010. The Rickettsia conorii autotransporter protein Sca1 promotes adherence to nonphagocytic mammalian cells. Infect Immun 78:1895–1904. doi:10.1128/IAI.01165-09 20176791 PMC2863548

[22] Cardwell MM, Martinez JJ. 2009. The Sca2 autotransporter protein from Rickettsia conorii is sufficient to mediate adherence to and invasion of cultured mammalian cells. Infect Immun 77:5272–5280. doi:10.1128/IAI.00201-09 19805531 PMC2786473

[23] Vellaiswamy M, Kowalczewska M, Merhej V, Nappez C, Vincentelli R, Renesto P, Raoult D. 2011. Characterization of rickettsial adhesin Adr2 belonging to a new group of adhesins in α-proteobacteria. Microb Pathog 50:233–242. doi:10.1016/j.micpath.2011.01.009 21288480

[24] Martinez JJ, Cossart P. 2004. Early signaling events involved in the entry of Rickettsia conorii into mammalian cells. J Cell Sci 117:5097–5106. doi:10.1242/jcs.01382 15383620

[25] Reed SCO, Serio AW, Welch MD. 2012. Rickettsia parkeri invasion of diverse host cells involves an Arp2/3 complex, WAVE complex and Rho-family GTPase-dependent pathway. Cell Microbiol 14:529–545. doi:10.1111/j.1462-5822.2011.01739.x 22188208 PMC3302968

[26] Welch MD, Rosenblatt J, Skoble J, Portnoy DA, Mitchison TJ. 1998. Interaction of human Arp2/3 complex and the Listeria monocytogenes ActA protein in actin filament nucleation. Science 281:105–108. doi:10.1126/science.281.5373.105 9651243

[27] Mauricio RPM, Jeffries CM, Svergun DI, Deane JE. 2017. The Shigella virulence factor IcsA relieves N-WASP autoinhibition by displacing the verprolin homology/cofilin/acidic (VCA) domain. J Biol Chem 292:134–145. doi:10.1074/jbc.M116.758003 27881679 PMC5217673

[28] Gouin E, Egile C, Dehoux P, Villiers V, Adams J, Gertler F, Li R, Cossart P. 2004. The RickA protein of Rickettsia conorii activates the Arp2/3 complex. Natur 427:451–467. doi:10.1038/nature02318 14749835

[29] Balraj P, Nappez C, Raoult D, Renesto P. 2008. Western-blot detection of RickA within spotted fever group rickettsiae using a specific monoclonal antibody. FEMS Microbiol Lett 286:257–262. doi:10.1111/j.1574-6968.2008.01283.x 18657112

[30] Haglund CM, Choe JE, Skau CT, Kovar DR, Welch MD. 2010. Rickettsia Sca2 is a bacterial formin-like mediator of actin-based motility. Nat Cell Biol 12:1057–1063. doi:10.1038/ncb2109 20972427 PMC3136050

[31] Kleba B, Clark TR, Lutter EI, Ellison DW, Hackstadt T. 2010. Disruption of the Rickettsia rickettsii Sca2 autotransporter inhibits actin-based motility. Infect Immun 78:2240–2247. doi:10.1128/IAI.00100-10 20194597 PMC2863521

[32] Rennoll-Bankert KE, Rahman MS, Guillotte ML, Lehman SS, Beier-Sexton M, Gillespie JJ, Azad AF. 2016. RalF-mediated activation of Arf6 controls Rickettsia typhi invasion by co-opting phosphoinositol metabolism. Infect Immun 84:3496–3506. doi:10.1128/IAI.00638-16 27698019 PMC5116726

[33] Rennoll-Bankert KE, Rahman MS, Gillespie JJ, Guillotte ML, Kaur SJ, Lehman SS, Beier-Sexton M, Azad AF. 2015. Which way in? the RalF Arf-GEF orchestrates Rickettsia host cell invasion. PLoS Pathog 11:e1005115. doi:10.1371/journal.ppat.1005115 26291822 PMC4546372

[34] Honda A, Nogami M, Yokozeki T, Yamazaki M, Nakamura H, Watanabe H, Kawamoto K, Nakayama K, Morris AJ, Frohman MA, Kanaho Y. 1999. Phosphatidylinositol 4-phosphate 5-kinase alpha is a downstream effector of the small G protein ARF6 in membrane ruffle formation. Cell 99:521–532. doi:10.1016/s0092-8674(00)81540-8 10589680

[35] Croisé P, Estay-Ahumada C, Gasman S, Ory S. 2014. Rho GTPases, phosphoinositides, and actin: a tripartite framework for efficient vesicular trafficking. Small GTPases 5:e29469. doi:10.4161/sgtp.29469 24914539 PMC4114633

[36] Voss OH, Gillespie JJ, Lehman SS, Rennoll SA, Beier-Sexton M, Rahman MS, Azad AF. 2020. Risk1, a phosphatidylinositol 3-kinase effector, promotes Rickettsia typhi intracellular survival. mBio 11:1–22. doi:10.1128/mBio.00820-20 PMC7298712 32546622

[37] Hilbi H. 2006. Modulation of phosphoinositide metabolism by pathogenic bacteria. Cell Microbiol 8:1697–1706. doi:10.1111/j.1462-5822.2006.00793.x 16939534

[38] Dong N, Niu M, Hu L, Yao Q, Zhou R, Shao F. 2016. Modulation of membrane phosphoinositide dynamics by the phosphatidylinositide 4-kinase activity of the Legionella LepB effector. Nat Microbiol 2:16236. doi:10.1038/nmicrobiol.2016.236 27941800

[39] Li G, Liu H, Luo ZQ, Qiu J. 2021. Modulation of phagosome phosphoinositide dynamics by a Legionella phosphoinositide 3-kinase. EMBO Rep 22:e51163. doi:10.15252/embr.202051163 33492731 PMC7926237

[40] Hsieh T-S, Lopez VA, Black MH, Osinski A, Pawłowski K, Tomchick DR, Liou J, Tagliabracci VS. 2021. Dynamic remodeling of host membranes by self-organizing bacterial effectors. Science 372:935–941. doi:10.1126/science.aay8118 33927055 PMC8543759

[41] Kim DJ, Park KS, Kim JH, Yang SH, Yoon JY, Han BG, Kim HS, Lee SJ, Jang JY, Kim KH, Kim MJ, Song JS, Kim HJ, Park CM, Lee SK, Lee BI, Suh SW. 2010. Helicobacter pylori proinflammatory protein up-regulates NF-kappaB as a cell-translocating Ser/Thr kinase. Proc Natl Acad Sci USA 107:21418–21423. doi:10.1073/pnas.1010153107 21098302 PMC3003084

[42] Edgar RC. 2004. MUSCLE: multiple sequence alignment with high accuracy and high throughput. Nucleic Acids Res 32:1792–1797. doi:10.1093/nar/gkh340 15034147 PMC390337

[43] Wood DO, Azad AF. 2000. Genetic manipulation of rickettsiae: a preview. Infect Immun 68:6091–6093. doi:10.1128/IAI.68.11.6091-6093.2000 11035710 PMC97684

[44] Ledvina HE, Kelly KA, Eshraghi A, Plemel RL, Peterson SB, Lee B, Steele S, Adler M, Kawula TH, Merz AJ, Skerrett SJ, Celli J, Mougous JD. 2018. A phosphatidylinositol 3-kinase effector alters phagosomal maturation to promote intracellular growth of Francisella. Cell Host & Microbe 24:285–295. doi:10.1016/j.chom.2018.07.003 30057173 PMC6394229

[45] Park SM, Ohkuma M, Masuda Y, Ohta A, Takagi M. 1997. Galactose-inducible expression systems in Candida maltosa using promoters of newly-isolated GAL1 and GAL10 genes. Yeast 13:21–29. doi:10.1002/(SICI)1097-0061(199701)13:1<21::AID-YEA58>3.0.CO;2-L 9046083

[46] Gillespie J.J, Kaur SJ, Rahman MS, Rennoll-Bankert K, Sears KT, Beier-Sexton M, Azad AF. 2015. Secretome of obligate intracellular Rickettsia. FEMS Microbiol Rev 39:47–80. doi:10.1111/1574-6976.12084 25168200 PMC4344940

[47] Gillespie J.J, Ammerman NC, Dreher-Lesnick SM, Rahman MS, Worley MJ, Setubal JC, Sobral BS, Azad AF. 2009. An anomalous type IV secretion system in Rickettsia is evolutionarily conserved. PLoS One 4:e4833. doi:10.1371/journal.pone.0004833 19279686 PMC2653234

[48] Li YG, Christie PJ. 2018. The Agrobacterium VirB/VirD4 T4SS: mechanism and architecture defined through in vivo mutagenesis and chimeric systems. Curr Top Microbiol Immunol 418:233–260. doi:10.1007/82_2018_94 29808338 PMC7011205

[49] Gillespie Joseph J, Phan IQH, Driscoll TP, Guillotte ML, Lehman SS, Rennoll-Bankert KE, Subramanian S, Beier-Sexton M, Myler PJ, Rahman MS, Azad AF. 2016. The Rickettsia type IV secretion system: unrealized complexity mired by gene family expansion. Pathog Dis 74:ftw058. doi:10.1093/femspd/ftw058 27307105 PMC5505475

[50] Llosa M, Alkorta I. 2017. Coupling proteins in type IV secretion. Curr Top Microbiol Immunol 413:143–168. doi:10.1007/978-3-319-75241-9_6 29536358

[51] Costa TRD, Harb L, Khara P, Zeng L, Hu B, Christie PJ. 2021. Type IV secretion systems: advances in structure, function, and activation. Mol Microbiol 115:436–452. doi:10.1111/mmi.14670 33326642 PMC8026593

[52] Atmakuri K, Ding Z, Christie PJ. 2003. VirE2, a type IV secretion substrate, interacts with the VirD4 transfer protein at cell poles of Agrobacterium tumefaciens. Mol Microbiol 49:1699–1713. doi:10.1046/j.1365-2958.2003.03669.x 12950931 PMC3882298

[53] Vergunst AC, van Lier MCM, den Dulk-Ras A, Stüve TAG, Ouwehand A, Hooykaas PJJ. 2005. Positive charge is an important feature of the C-terminal transport signal of the VirB/D4-translocated proteins of Agrobacterium. Proc Natl Acad Sci USA 102:832–837. doi:10.1073/pnas.0406241102 15644442 PMC545537

[54] Lehman SS, Noriea NF, Aistleitner K, Clark TR, Dooley CA, Nair V, Kaur SJ, Rahman MS, Gillespie JJ, Azad AF, Hackstadt T. 2018. The rickettsial ankyrin repeat protein 2 is a type IV secreted effector that associates with the endoplasmic reticulum. mBio 9:e00975-18. doi:10.1128/mBio.00975-18 29946049 PMC6020290

[55] Karimova G, Pidoux J, Ullmann A, Ladant D. 1998. A bacterial two-hybrid system based on a reconstituted signal transduction pathway. Proc Natl Acad Sci USA 95:5752–5756. doi:10.1073/pnas.95.10.5752 9576956 PMC20451

[56] Karimova G, Ullmann A, Ladant D. 2000. Bordetella pertussis adenylate cyclase toxin as a tool to analyze molecular interactions in a bacterial two-hybrid system. Int J Med Microbiol 290:441–445. doi:10.1016/S1438-4221(00)80060-0 11111924

[57] Luo ZQ, Isberg RR. 2004. Multiple substrates of the Legionella pneumophila Dot/Icm system identified by interbacterial protein transfer. Proc Natl Acad Sci U S A 101:841–846. doi:10.1073/pnas.0304916101 14715899 PMC321768

[58] Berger KH, Isberg RR. 1993. Two distinct defects in intracellular growth complemented by a single genetic locus in Legionella pneumophila. Mol Microbiol 7:7–19. doi:10.1111/j.1365-2958.1993.tb01092.x 8382332

[59] Berger KH, Merriam JJ, Isberg RR. 1994. Altered intracellular targeting properties associated with mutations in the Legionella pneumophila dotA gene. Mol Microbiol 14:809–822. doi:10.1111/j.1365-2958.1994.tb01317.x 7891566

[60] Nagai H, Kagan JC, Zhu X, Kahn RA, Roy CR. 2002. A bacterial guanine nucleotide exchange factor activates ARF on Legionella phagosomes. Science 295:679–682. doi:10.1126/science.1067025 11809974

[61] Zhen X, Wu Y, Ge J, Fu J, Ye L, Lin N, Huang Z, Liu Z, Luo Z-Q, Qiu J, Ouyang S. 2022. Molecular mechanism of toxin neutralization in the HipBST toxin-antitoxin system of Legionella pneumophila. Nat Commun 13:4333. doi:10.1038/s41467-022-32049-x 35882877 PMC9325769

[62] Gonzalez-Sastre F, Folch-Pi J. 1968. Thin-layer chromatography of the phosphoinositides. J Lipid Res 9:532–533. doi:10.1016/S0022-2275(20)42734-8 4302257

[63] Taylor GS, Maehama T, Dixon JE. 2000. Myotubularin, a protein tyrosine phosphatase mutated in myotubular myopathy, dephosphorylates the lipid second messenger, phosphatidylinositol 3-phosphate. Proc Natl Acad Sci USA 97:8910–8915. doi:10.1073/pnas.160255697 10900271 PMC16795

[64] Konrad G, Schlecker T, Faulhammer F, Mayinger P. 2002. Retention of the yeast Sac1p phosphatase in the endoplasmic reticulum causes distinct changes in cellular phosphoinositide levels and stimulates microsomal ATP transport. J Biol Chem 277:10547–10554. doi:10.1074/jbc.M200090200 11792713

[65] Pagliarini DJ, Worby CA, Dixon JE. 2004. A PTEN-like phosphatase with a novel substrate specificity. J Biol Chem 279:38590–38596. doi:10.1074/jbc.M404959200 15247229

[66] Foti M, Audhya A, Emr SD. 2001. Sac1 lipid phosphatase and Stt4 phosphatidylinositol 4-kinase regulate a pool of phosphatidylinositol 4-phosphate that functions in the control of the actin cytoskeleton and vacuole morphology. Mol Biol Cell 12:2396–2411. doi:10.1091/mbc.12.8.2396 11514624 PMC58602

[67] Gaullier JM, Simonsen A, D’Arrigo A, Bremnes B, Stenmark H, Aasland R. 1998. FYVE fingers bind PtdIns(3)P. Nature 394:432–433. doi:10.1038/28767 9697764

[68] Stahelin RV, Long F, Diraviyam K, Bruzik KS, Murray D, Cho W. 2002. Phosphatidylinositol 3-phosphate induces the membrane penetration of the FYVE domains of Vps27p and Hrs. J Biol Chem 277:26379–26388. doi:10.1074/jbc.M201106200 12006563

[69] Guittard G, Gérard A, Dupuis-Coronas S, Tronchère H, Mortier E, Favre C, Olive D, Zimmermann P, Payrastre B, Nunès JA. 2009. Cutting edge: Dok-1 and Dok-2 adaptor molecules are regulated by phosphatidylinositol 5-phosphate production in T cells. J Immunol 182:3974–3978. doi:10.4049/jimmunol.0804172 19299694

[70] He J, Scott JL, Heroux A, Roy S, Lenoir M, Overduin M, Stahelin RV, Kutateladze TG. 2011. Molecular basis of phosphatidylinositol 4-phosphate and ARF1 GTPase recognition by the FAPP1 pleckstrin homology (PH) domain. J Biol Chem 286:18650–18657. doi:10.1074/jbc.M111.233015 21454700 PMC3099681

[71] Liu Y, Kahn RA, Prestegard JH. 2014. Interaction of Fapp1 with Arf1 and PI4P at a membrane surface: an example of coincidence detection. Structure 22:421–430. doi:10.1016/j.str.2013.12.011 24462251 PMC3951685

[72] Walker EH, Pacold ME, Perisic O, Stephens L, Hawkins PT, Wymann MP, Williams RL. 2000. Structural determinants of phosphoinositide 3-kinase inhibition by wortmannin, LY294002, quercetin, myricetin, and staurosporine. Mol Cell 6:909–919. doi:10.1016/s1097-2765(05)00089-4 11090628

[73] Huang J, Brumell JH. 2014. Bacteria-autophagy interplay: a battle for survival. Nat Rev Microbiol 12:101–114. doi:10.1038/nrmicro3160 24384599 PMC7097477

[74] He C, Levine B. 2010. The Beclin 1 interactome. Curr Opin Cell Biol 22:140–149. doi:10.1016/j.ceb.2010.01.001 20097051 PMC2854269

[75] Hsu F, Zhu W, Brennan L, Tao L, Luo Z-Q, Mao Y. 2012. Structural basis for substrate recognition by a unique Legionella phosphoinositide phosphatase. Proc Natl Acad Sci U S A 109:13567–13572. doi:10.1073/pnas.1207903109 22872863 PMC3427105

[76] Weber SS, Ragaz C, Reus K, Nyfeler Y, Hilbi H. 2006. Legionella pneumophila exploits PI(4)P to anchor secreted effector proteins to the replicative vacuole. PLoS Pathog 2:e46. doi:10.1371/journal.ppat.0020046 16710455 PMC1463015

[77] Qiu J, Luo ZQ. 2017. Legionella and Coxiella effectors: strength in diversity and activity. Nat Rev Microbiol 15:591–605. doi:10.1038/nrmicro.2017.67 28713154

[78] Wymann MP, Bulgarelli-Leva G, Zvelebil MJ, Pirola L, Vanhaesebroeck B, Waterfield MD, Panayotou G. 1996. Wortmannin inactivates phosphoinositide 3-kinase by covalent modification of Lys-802, a residue involved in the phosphate transfer reaction. Mol Cell Biol 16:1722–1733. doi:10.1128/MCB.16.4.1722 8657148 PMC231159

[79] Furuya T, Kim M, Lipinski M, Li J, Kim D, Lu T, Shen Y, Rameh L, Yankner B, Tsai LH, Yuan J. 2010. Negative regulation of Vps34 by Cdk mediated phosphorylation. Mol Cell 38:500–511. doi:10.1016/j.molcel.2010.05.009 20513426 PMC2888511

[80] Chong A, Wehrly TD, Child R, Hansen B, Hwang S, Virgin HW, Celli J. 2012. Cytosolic clearance of replication-deficient mutants reveals Francisella tularensis interactions with the autophagic pathway. Autophagy 8:1342–1356. doi:10.4161/auto.20808 22863802 PMC3442881

[81] Gutierrez MG, Master SS, Singh SB, Taylor GA, Colombo MI, Deretic V. 2004. Autophagy is a defense mechanism inhibiting BCG and Mycobacterium tuberculosis survival in infected macrophages. Cell 119:753–766. doi:10.1016/j.cell.2004.11.038 15607973

[82] Engström P, Burke TP, Mitchell G, Ingabire N, Mark KG, Golovkine G, Iavarone AT, Rape M, Cox JS, Welch MD. 2019. Evasion of autophagy mediated by Rickettsia surface protein OmpB is critical for virulence. Nat Microbiol 4:2538–2551. doi:10.1038/s41564-019-0583-6 31611642 PMC6988571

[83] Yoshikawa Y, Ogawa M, Hain T, Chakraborty T, Sasakawa C. 2009. Listeria monocytogenes ActA is a key player in evading autophagic recognition. Autophagy 5:1220–1221. doi:10.4161/auto.5.8.10177 19855178

[84] Choy A, Dancourt J, Mugo B, O’Connor TJ, Isberg RR, Melia TJ, Roy CR. 2012. The Legionella effector RavZ inhibits host autophagy through irreversible Atg8 deconjugation. Science 338:1072–1076. doi:10.1126/science.1227026 23112293 PMC3682818

[85] Xu Y, Zhou P, Cheng S, Lu Q, Nowak K, Hopp AK, Li L, Shi X, Zhou Z, Gao W, Li D, He H, Liu X, Ding J, Hottiger MO, Shao F. 2019. A bacterial effector reveals the V-ATPase-ATG16L1 axis that initiates xenophagy. Cell 178:552–566. doi:10.1016/j.cell.2019.06.007 31327526

[86] Martinez E, Allombert J, Cantet F, Lakhani A, Yandrapalli N, Neyret A, Norville IH, Favard C, Muriaux D, Bonazzi M. 2016. Coxiella burnetii effector CvpB modulates phosphoinositide metabolism for optimal vacuole development. Proc Natl Acad Sci U S A 113:E3260–9. doi:10.1073/pnas.1522811113 27226300 PMC4988616

[87] Siadous FA, Cantet F, Van Schaik E, Burette M, Allombert J, Lakhani A, Bonaventure B, Goujon C, Samuel J, Bonazzi M, Martinez E. 2021. Coxiella effector protein CvpF subverts RAB26-dependent autophagy to promote vacuole biogenesis and virulence. Autophagy 17:706–722. doi:10.1080/15548627.2020.1728098 32116095 PMC8032239

[88] Niu H, Xiong Q, Yamamoto A, Hayashi-Nishino M, Rikihisa Y. 2012. Autophagosomes induced by a bacterial Beclin 1 binding protein facilitate obligatory intracellular infection. Proc Natl Acad Sci USA 109:20800–20807. doi:10.1073/pnas.1218674109 23197835 PMC3529060

[89] Lin M, Liu H, Xiong Q, Niu H, Cheng Z, Yamamoto A, Rikihisa Y. 2016. Ehrlichia secretes Etf-1 to induce autophagy and capture nutrients for its growth through RAB5 and class III phosphatidylinositol 3-kinase. Autophagy 12:2145–2166. doi:10.1080/15548627.2016.1217369 27541856 PMC5103349

[90] Bechelli J, Rumfield CS, Walker DH, Widen S, Khanipov K, Fang R. 2021. Subversion of host innate immunity by Rickettsia australis via a modified autophagic response in macrophages. Front Immunol 12:638469. doi:10.3389/fimmu.2021.638469 33912163 PMC8071864

[91] Bechelli J, Vergara L, Smalley C, Buzhdygan TP, Bender S, Zhang W, Liu Y, Popov VL, Wang J, Garg N, Hwang S, Walker DH, Fang R. 2019. Atg5 supports Rickettsia australis infection in macrophages in vitro and in vivo. Infect Immun 87. doi:10.1128/IAI.00651-18 PMC6300621 30297526

[92] Voss OH, Gaytan H, Ullah S, Sadik M, Moin I, Rahman MS, Azad AF. 2023. Autophagy facilitates intracellular survival of pathogenic rickettsiae in macrophages via evasion of autophagosomal maturation and reduction of microbicidal pro-inflammatory IL-1 cytokine responses. Microbiol Spectr 11:e0279123. doi:10.1128/spectrum.02791-23 37819111 PMC10715094

[93] Engström P, Burke TP, Tran CJ, Iavarone AT, Welch MD. 2021. Lysine methylation shields an intracellular pathogen from ubiquitylation and autophagy. Sci Adv 7:eabg2517. doi:10.1126/sciadv.abg2517 34172444 PMC8232902

[94] Yang DCH, Abeykoon AH, Choi B-E, Ching W-M, Chock PB. 2017. Outer membrane protein OmpB methylation may mediate bacterial virulence. Trends Biochem Sci 42:936–945. doi:10.1016/j.tibs.2017.09.005 29037863

[95] Abeykoon A, Wang G, Chao C-C, Chock PB, Gucek M, Ching W-M, Yang DCH. 2014. Multimethylation of Rickettsia OmpB catalyzed by lysine methyltransferases. J Biol Chem 289:7691–7701. doi:10.1074/jbc.M113.535567 24497633 PMC3953280

[96] Lamason RL, Bastounis E, Kafai NM, Serrano R, del Álamo JC, Theriot JA, Welch MD. 2016. Rickettsia Sca4 reduces vinculin-mediated intercellular tension to promote spread. Cell 167:670–683. doi:10.1016/j.cell.2016.09.023 27768890 PMC5097866

[97] Borgo GM, Burke TP, Tran CJ, Lo NTN, Engström P, Welch MD. 2022. A patatin-like phospholipase mediates Rickettsia parkeri escape from host membranes. Nat Commun 13:3656. doi:10.1038/s41467-022-31351-y 35760786 PMC9237051

[98] Driskell LO, Yu X, Zhang L, Liu Y, Popov VL, Walker DH, Tucker AM, Wood DO. 2009. Directed mutagenesis of the Rickettsia prowazekii pld gene encoding phospholipase D. Infect Immun 77:3244–3248. doi:10.1128/IAI.00395-09 19506016 PMC2715659

[99] Gong W, Wang P, Xiong X, Jiao J, Yang X, Wen B. 2015. Enhanced protection against Rickettsia rickettsii infection in C3H/HeN mice by immunization with a combination of a recombinant adhesin rAdr2 and a protein fragment rOmpB-4 derived from outer membrane protein B. Vaccine (Auckl) 33:985–992. doi:10.1016/j.vaccine.2015.01.017 25597943

[100] Alhassan A, Liu H, McGill J, Cerezo A, Jakkula LUMR, Nair ADS, Winkley E, Olson S, Marlow D, Sahni A, Narra HP, Sahni S, Henningson J, Ganta RR. 2019. Rickettsia rickettsii whole-cell antigens offer protection against rocky mountain spotted fever in the canine host. Infect Immun 87. doi:10.1128/IAI.00628-18 PMC6346123 30396898

[101] Duan C, Meng Y, Wang X, Xiong X, Wen B. 2011. Exploratory study on pathogenesis of far-eastern spotted fever. Am J Trop Med Hyg 85:504–509. doi:10.4269/ajtmh.2011.10-0660 21896812 PMC3163874

[102] Weinberg EH, Stakebake JR, Gerone PJ. 1969. Plaque assay for Rickettsia rickettsii. J Bacteriol 98:398–402. doi:10.1128/jb.98.2.398-402.1969 4977475 PMC284828

[103] Ma K, Shu R, Liu H, Ge J, Liu J, Lu Q, Fu J, Liu X, Qiu J. 2024. Legionella effectors SidC/SdcA ubiquitinate multiple small GTPases and SNARE proteins to promote phagosomal maturation. Cell Mol Life Sci 81. doi:10.1007/s00018-024-05271-7 PMC11335287 38836877

[104] Song L, Xie Y, Li C, Wang L, He C, Zhang Y, Yuan J, Luo J, Liu X, Xiu Y, Li H, Gritsenko M, Nakayasu ES, Feng Y, Luo Z-Q. 2021. The Legionella effector SdjA is a bifunctional enzyme that distinctly regulates phosphoribosyl ubiquitination. mBio 12:e0231621. doi:10.1128/mBio.02316-21 34488448 PMC8546864

[105] O’Shea EK, Klemm JD, Kim PS, Alber T. 1991. X-ray structure of the GCN4 leucine zipper, a two-stranded, parallel coiled coil. Science 254:539–544. doi:10.1126/science.1948029 1948029

[106] Karimova G, Ullmann A, Ladant D. 2000. A bacterial two-hybrid system that exploits a cAMP signaling cascade in Escherichia coli. Methods Enzymol 328:59–73. doi:10.1016/s0076-6879(00)28390-0 11075338

[107] McCloskey A, Perri K, Chen T, Han A, Luo Z-Q. 2021. The metaeffector MesI regulates the activity of the Legionella effector SidI through direct protein-protein interactions. Microbes Infect 23:104794. doi:10.1016/j.micinf.2021.104794 33571674 PMC9406241

[108] Charpentier X, Gabay JE, Reyes M, Zhu JW, Weiss A, Shuman HA. 2009. Chemical genetics reveals bacterial and host cell functions critical for type IV effector translocation by Legionella pneumophila. PLoS Pathog 5:e1000501. doi:10.1371/journal.ppat.1000501 19578436 PMC2698123

[109] Song L, Luo J, Wang H, Huang D, Tan Y, Liu Y, Wang Y, Yu K, Zhang Y, Liu X, Li D, Luo ZQ. 2022. Legionella pneumophila regulates host cell motility by targeting Phldb2 with a 14-3-3ζ-dependent protease effector. Elife 11:e73220. doi:10.7554/eLife.73220 35175192 PMC8871388

[110] Thomas BJ, Rothstein R. 1989. Elevated recombination rates in transcriptionally active DNA. Cell 56:619–630. doi:10.1016/0092-8674(89)90584-9 2645056

[111] Gietz RD, Schiestl RH, Willems AR, Woods RA. 1995. Studies on the transformation of intact yeast cells by the LiAc/SS‐DNA/PEG procedure. Yeast 11:355–360. doi:10.1002/yea.320110408 7785336

[112] Szymanski EP, Kerscher O. 2013. Budding yeast protein extraction and purification for the study of function, interactions, and post-translational modifications. J Vis Exp. doi:10.3791/50921:e50921 PMC3968972 24300101

