# Supplementary figures and images for: An effector of phosphatidylinositol 3-kinase activity promotes Rickettsia rickettsii virulence by enhancing autophagy
Source: mBio. 2025 Sep 22;16(11):e02284-25. doi: 10.1128/mbio.02284-25 (PMC12607708; doi:10.1128/mbio.02284-25)

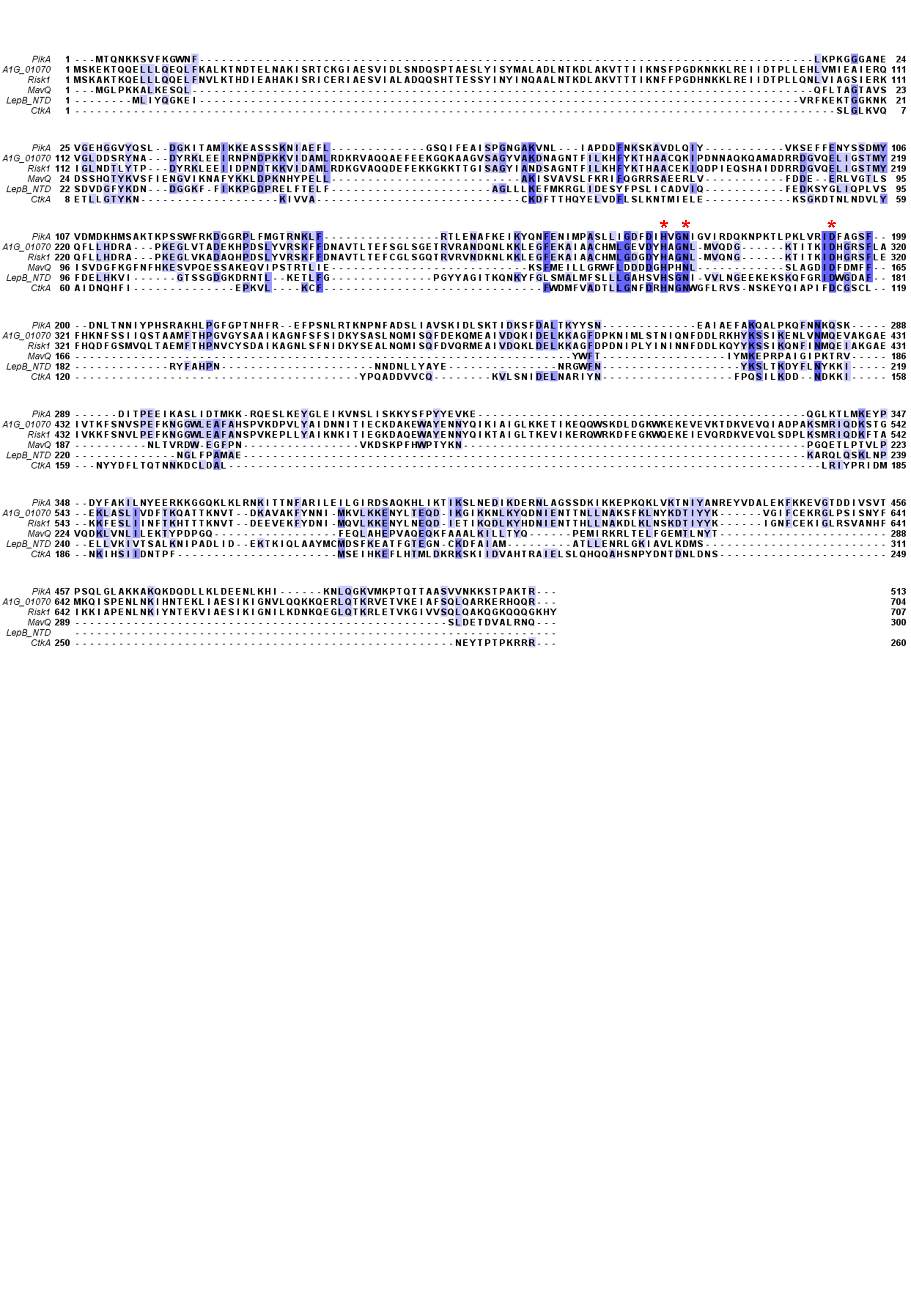

Supplement: Figure S1 — The H-N-D motif of PikA is conserved in many bacterial effectors with PI kinase activity. [file mbio.02284-25-s0001.tif]

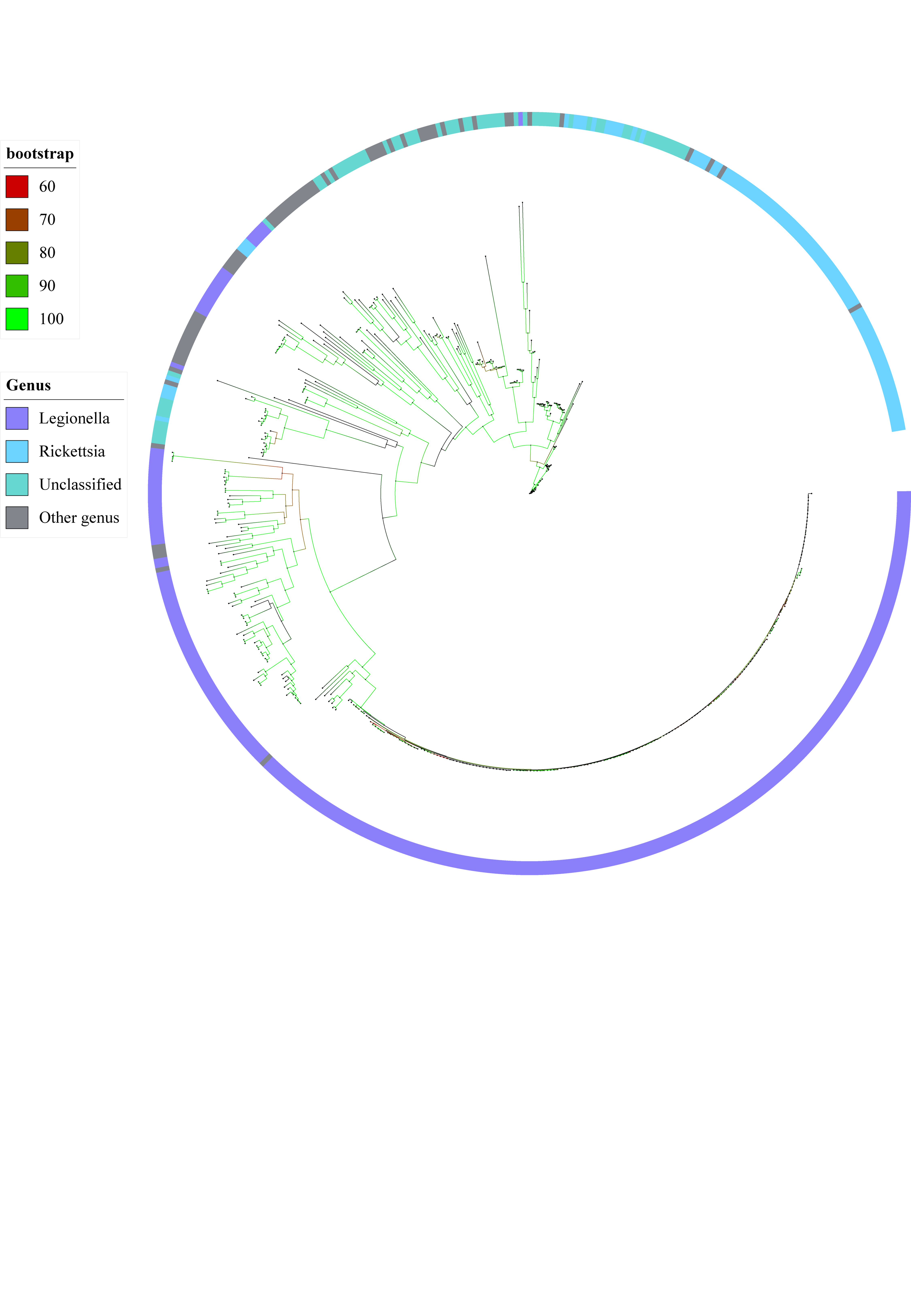

Supplement: Figure S2 — Distribution of PikA homologs in different groups of Rickettsia and Legionella species. [file mbio.02284-25-s0002.tif]

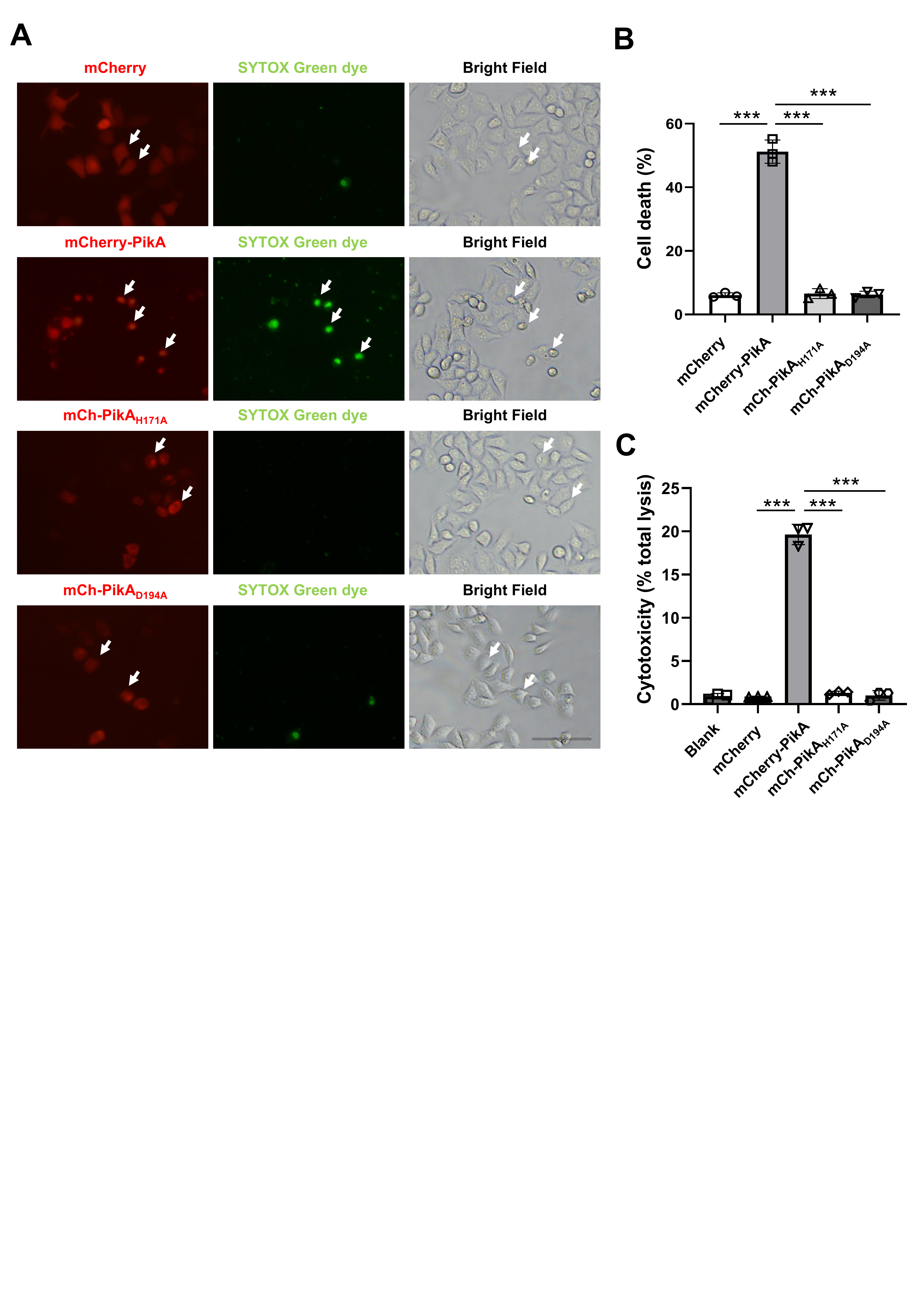

Supplement: Figure S3 — Expression of PikA caused mammalian cell death. [file mbio.02284-25-s0003.tif]

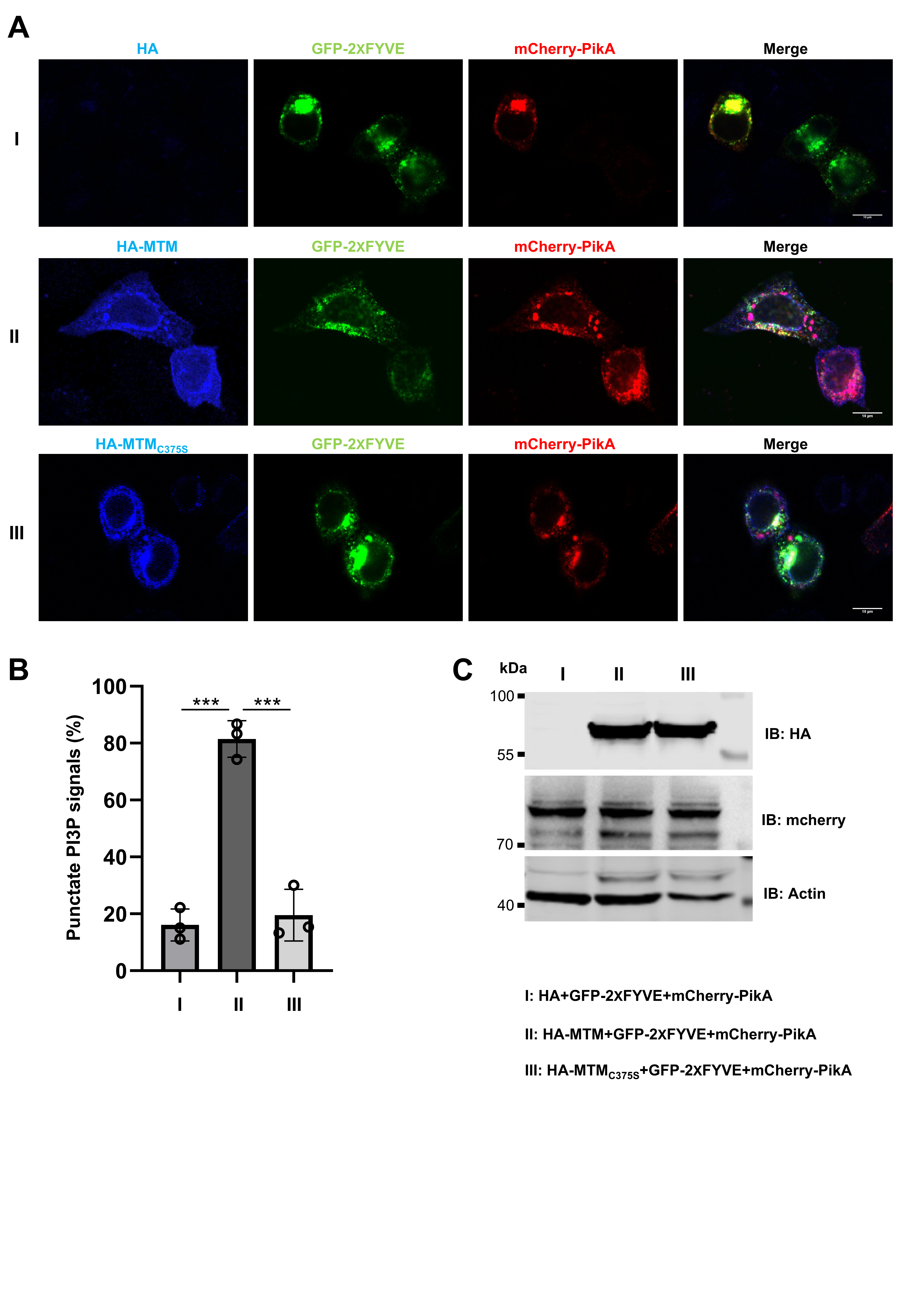

Supplement: Figure S4 — MTM reverses PikA-triggered clustering of PI3P signals to vesicle-like distribution. [file mbio.02284-25-s0004.tif]

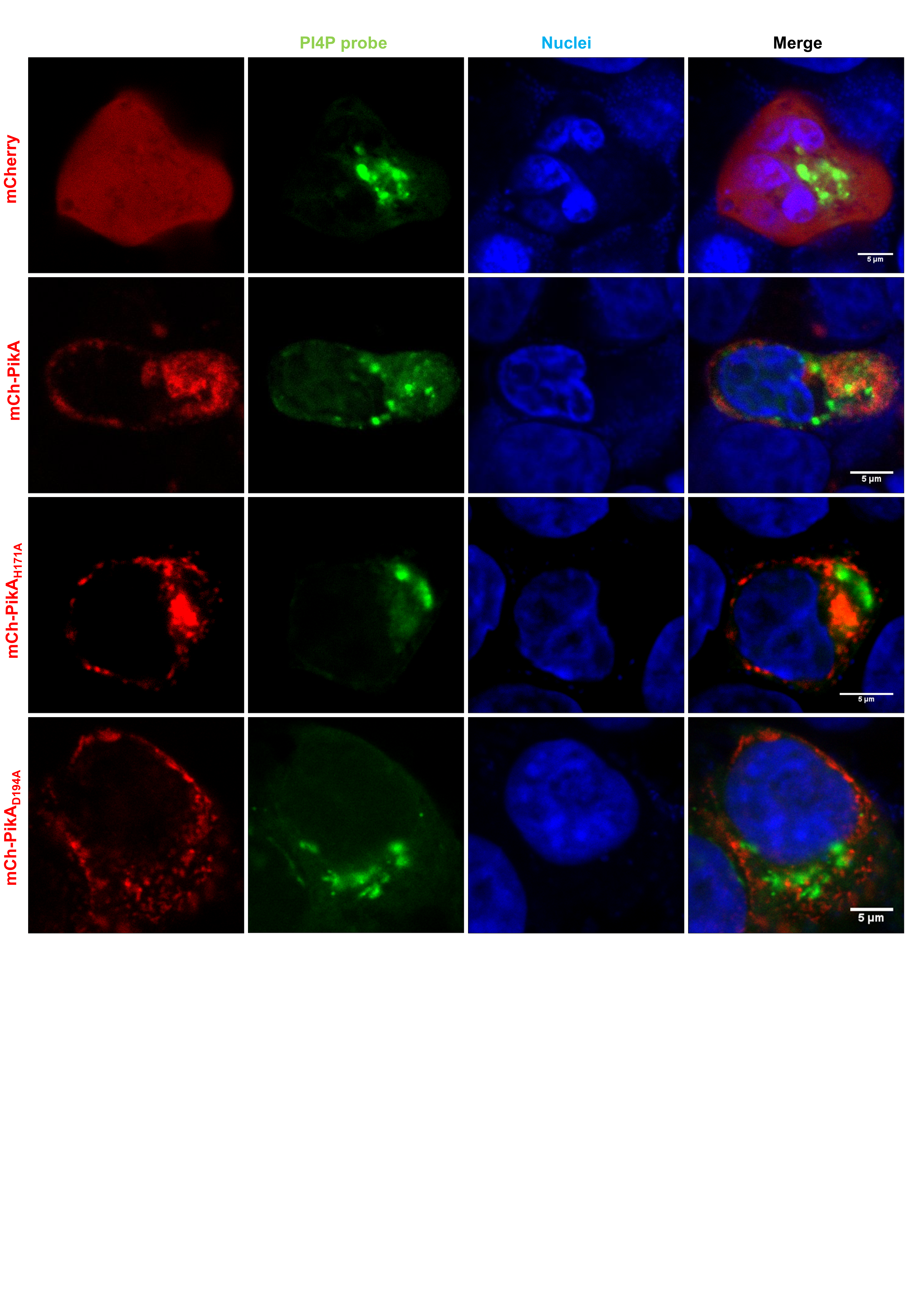

Supplement: Figure S5 — PikA does not influence the distribution of PI4P. [file mbio.02284-25-s0005.tif]

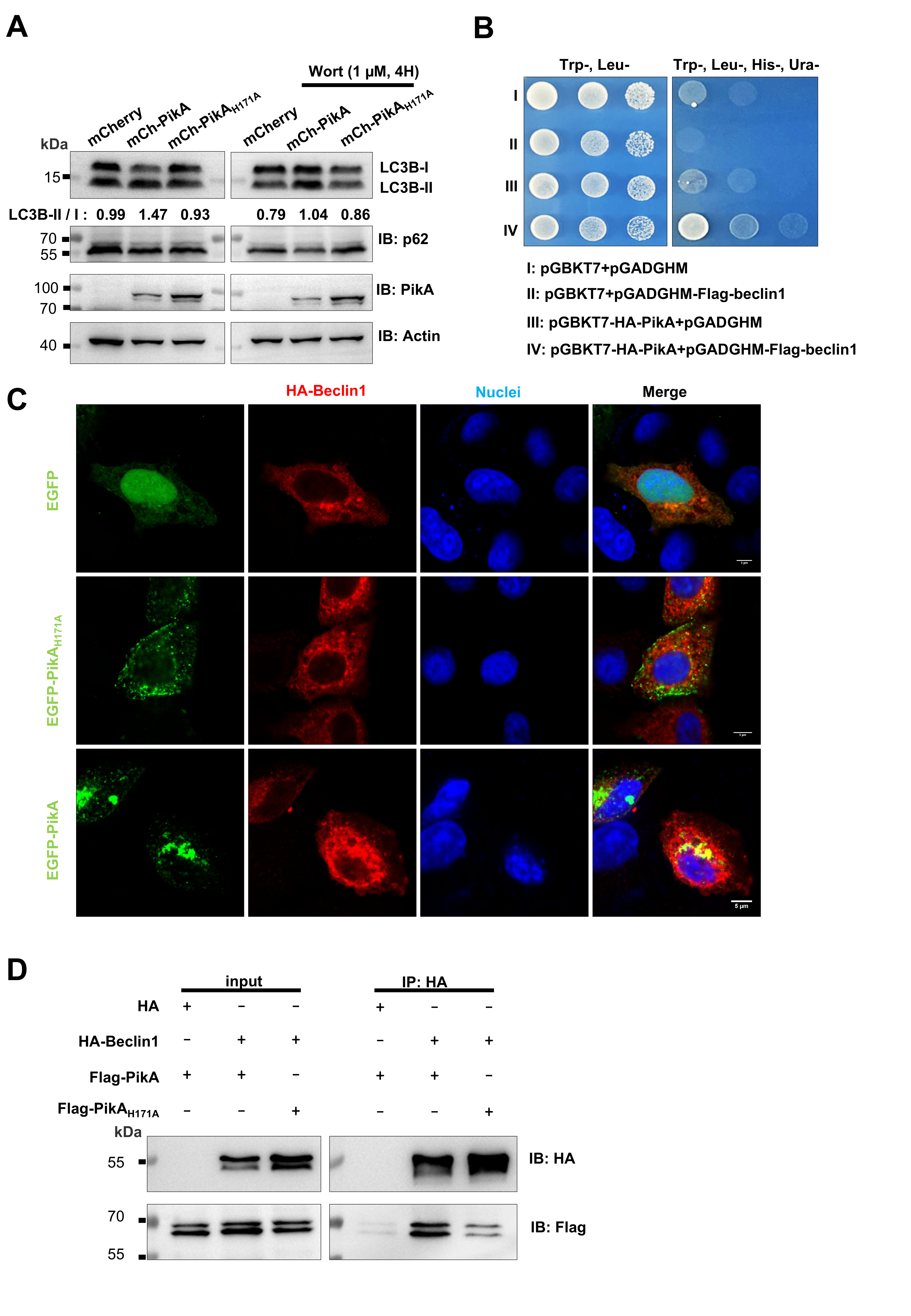

Supplement: Figure S6 — Kinase activity-dependent interaction between PikA and Beclin1. [file mbio.02284-25-s0006.tif]
