# Supplementary material for: An effector of phosphatidylinositol 3-kinase activity promotes Rickettsia rickettsii virulence by enhancing autophagy
Source: mBio. 2025 Sep 22;16(11):e02284-25. doi: 10.1128/mbio.02284-25 (PMC12607708; doi:10.1128/mbio.02284-25)
Supplement: Legends — for Fig. S1-S6. [file mbio.02284-25-s0007.docx]

**Legends for Supplementary Data**

**Fig. S1 The H-N-D motif of PikA is conserved in many bacterial effectors with PI kinase activity**

Multiple sequence alignment of PikA with *A1G_01070* and kinases from different bacterial pathogens by MUSCLE. The H-N-D residues critical for catalysis and activation in blue background were indicated by an asterisk. The numbers at the ends of the sequences indicate the positions of the residues in the proteins.

**Fig. S2 Distribution of PikA homologs in different groups of *Rickettsia* and *Legionella* species**

Homologs of PikA were retrieved and the phylogenetic tree was generated by three rounds of Position-Specific Iterated BLAST (PSI-BLAST) of *A1G_04485*.

**Fig. S3 Expression of PikA caused mammalian cell death**

**A.** Representative fluorescence and bright field images of HeLa cells transfected to express mCherry, mCherry-tagged PikA or its enzymatically inactive mutants. Cells transfected for 18 h were used for staining SYTOX green in Hanks buffer for 15 min before image acquisition. Scale bars, 100 µm.

**B.** Quantitation of cells with both red and green fluorescence in samples transfected to express PikA or its mutants. For each sample, at least 300 cells were scored and results (mean±s. e.) shown were from three independent experiments.

**C.** LDH release assays by HeLa cells expressing PikA and its mutants. Cells transfected to express the indicated alleles of PikA were measured for LDH in culture supernatant. Ratio was calculated using cells lysed by a detergent as max release. Shown in C are percentages of cell death as mean±s. e. from three independent experiments.

**Fig. S4 MTM reverses PikA-triggered clustering of PI3P signals to vesicle-like** **distribution**.

**A**. HeLa cells co-transfected to express HA-MTM or HA-MTM_C375S_ with mCherry-PikA and GFPxFYVE (PI3P probe). After 18 h, cells were fixed with 4% paraformaldehyde and subjected to immunoflurescene imaging. Scale bar, 10 µm.

**B**. Quantitation of cells exhibiting punctate PI3P signals simultaneously expressing mCherry-PikA and MTM or its mutants. For each sample, at least 300 cells were scored and results (mean±s. e.) shown were from three independent experiments.

**C**. The expression of mCherry-PikA , HA-tagged MTM and its mutant was detected by immunoblotting with the appropriate antibodies. Actin was detected as a loading control.

**Fig. S5 PikA does not influence the distribution of PI4P**

Representative fluorescence images of HeLa cells expressing the PI4P probe GFP-PH_FAPP1_ and mCherry-PikA or its mutants mCherry-PikA_H171A_, mcherry-PikA_D194A_. Note the scattered distribution patterns of GFP fluorescence signals among cells expressing these alleles of PikA. Bar, 5 µm.

**Fig. S6 Kinase activity-dependent interaction between PikA and Beclin1**

**A**. PikA-induced increase in the ratio of LC3B-II/I was insensitive to wortmannin. Lysates from HEK293T cells transfected with indicated plasmids for 14 h, wortmannin was added at 1 µM to a subset of samples prior to probing for LC3B, p62 and PikA by immunoblotting. Actin was probed as a loading control. Data shown were one representative of three independent experiments with similar results.

**B**. Interaction between PikA and Beclin1 determined by yeast two-hybrid assay. The yeast strain AH109 was transformed with pairs of the indicated plasmids, serially diluted cells were spotted on dropout medium to examine growth and interactions. Images were acquired after incubation for 4 d at 30°C. The growth of yeast on the Ura^-^/His^-^/Leu^-^/Trp^-^ dropout medium indicates positive interactions.

**C**. Colocalization between PikA and Beclin1 requires its kinase activity. HeLa cells were transfected to co-express HA-Beclin1 and GFP, GFP-PikA or GFP-PikA_H171A_ for 14 h. Fixed cells were immunostained with anti-HA antibody and a Texas Red-conjugated secondary antibody. Nuclei were stained by Hoechst. Images were acquired using a confocal microscope. Bar, 5 µm.

**D**. The interaction between PikA and Beclin1 requires its kinase activity. Lysates from HEK293T cells transfected with the indicated plasmid combinations for 14 h were immunoprecipitated with beads coated with HA antibody and the presence of precipitated proteins was detected by immunoblotting.
