# Supplementary material for: An effector of phosphatidylinositol 3-kinase activity promotes Rickettsia rickettsii virulence by enhancing autophagy
Source: mBio. 2025 Sep 22;16(11):e02284-25. doi: 10.1128/mbio.02284-25 (PMC12607708; doi:10.1128/mbio.02284-25)
Supplement: Table S1 — Bacterial strains, plasmids, and primers used in this study. [file mbio.02284-25-s0008.docx]

**Table S1 Bacterial strains, plasmids and primers used in this study**

**1. Bacterial strains**

| Bacterial Strains | Source | Identifier |
| --- | --- | --- |
| *L. pneumophila* (Philadelphia-1) Lp02 | (1) | N/A |
| *L. pneumophila* Lp03 | (1) | N/A |
| Lp02(pZLQ-flag-TEM1-PikA) | This study | N/A |
| Lp03(pZLQ-flag-TEM1-PikA) | This study | N/A |
| Lp02(pXDC61-TEM1-RaIF) | This study | N/A |
| Lp02(pXDC61-TEM1-Fab1) | This study | N/A |
| *E. coli* BTH101 | (2, 3) | N/A |
| BTH101 (pKT25, pUT18C-Flag) | This study | N/A |
| BTH101 (pKT25-zip, pUT18C-Flag-zip) | This study | N/A |
| BTH101 (pKT25, pUT18C-Flag-PikA) | This study | N/A |
| BTH101 (pKT25-Flag-PikA, pUT18C-Flag) | This study | N/A |
| BTH101 (pKT25-Flag-PikA, pUT18C-Flag-VirD4) | This study | N/A |
| *S. cerevisiae* W303 | 112 | N/A |
| W303(pYES2-3Flag) | This study | N/A |
| W303(pYES2-3Flag-PikA) | This study | N/A |
| W303(pYES2-3Flag- PikA_H171A_) | This study | N/A |
| W303(pYES2-3Flag- PikA_N174A_) | This study | N/A |
| W303(pYES2-3Flag PikA_D194A_) | This study | N/A |
| AH109 | MKBio | CAT# MF2356 |
| AH109 (pGBKT7, pGADGHM) | This study | N/A |
| AH109 (pGBKT7, pGADGHM-Flag-Beclin1) | This study | N/A |
| AH109 (pGBKT7-HA-PikA, pGADGHM) | This study | N/A |
| AH109 (pGBKT7-HA-PikA,pGADGHM-Flag-Beclin1) | This study | N/A |
| *E.coli* DH5α | TransGen | CAT# CD201 |
| *E.coli* BL21(DE3) | TransGen | CAT# CD601 |

**2. Plasmids**

| Plasmids | Source | Identifier |
| --- | --- | --- |
| pZLQ-flag::*TEM1*-*PikA* | This study | N/A |
| pXDC61::*TEM1-RaIF* | 61 | N/A |
| pXDC61::*TEM1*-*Fab1* | 61 | N/A |
| pKT25 | 55,56 | N/A |
| pKT25::*zip* | This study | N/A |
| pKT25::Flag-*PikA* | This study | N/A |
| pUT18C-Flag | This study | N/A |
| pUT18C-Flag::*zip* | This study | N/A |
| pUT18C-Flag::*VirD4* | This study | N/A |
| pYES2/CT | Invitrogen | CAT#V825120 |
| pYES2/CT:: 3×Flag | This study | N/A |
| pYES2/CT:: 3×Flag *PikA* | This study | N/A |
| pYES2/CT:: 3×Flag *PikA* *_H171A_* | This study | N/A |
| pYES2/CT:: 3×Flag *PikA* *_N174A_* | This study | N/A |
| pYES2/CT:: 3×Flag *PikA* *_D194A_* | This study | N/A |
| pmCherry-C1 | Clontech | CAT#632524 |
| pmCherry-C1:: *PikA* | This study | N/A |
| pmCherry-C1:: *PikA _H171A_* | This study | N/A |
| pmCherry-C1:: *PikA _D194A_* | This study | N/A |
| pET28a | Novagen | CAT#69864 |
| pET28a:: *PikA* | This study | N/A |
| pET28a:: *PikA _H171A_* | This study | N/A |
| pET28a:: *PikA _D194A_* | This study | N/A |
| pET-SUMO | Solarbio | VT008755 |
| pET-SUMO::*MTM1* | This study | N/A |
| pET-SUMO::*sac1p* | This study | N/A |
| pET-SUMO::*PLIP_△N68_* | This study | N/A |
| pEGFP-C1 | Clontech | CAT#6084-1 |
| pEGFP-C1:: *MTM1* | This study | N/A |
| pEGFP-C1:: *MTM1_C375S_* | This study | N/A |
| pEGFP-C1:: 2×FYVE | This study | N/A |
| pEGFP-C1::PH_FAPP1_ | This study | N/A |
| pEGFP-C1:: *PikA* | This study | N/A |
| pEGFP-C1:: *PikA _H171A_* | This study | N/A |
| pEGFP-C1:: *PikA _D194A_* | This study | N/A |
| pEGFP-N1 | Clontech | CAT#6085-1 |
| pEGFP-N1:: *Dok1* | This study | N/A |
| pAPH | (4) | N/A |
| pAPH:: *BECN1* | This study | N/A |
| pAPH:: *MTM1* | This study | N/A |
| pAPH:: *MTM1_C375S_* | This study | N/A |
| pCMV4×Flag | (5) | N/A |
| pCMV4×Flag:: *PikA* | This study | N/A |
| pGBKT7 | Clontech | CAT#630443 |
| pGBKT7:: HA-*PikA* | This study | N/A |
| pGADGH | Clontech | CAT#6182-1 |
| pGADGH:: Flag-*BECN1* | This study | N/A |

N/A, not applicable

**3. Primers**

| Primers | Sequence (Restriction enzyme sites are underlined) 5’-3’ | Note |
| --- | --- | --- |
| pHD1001 | cgcggatccatgacgcaaaataagaaatcagt | *PikA* *5F BamHI* |
| pHD1002 | acgcgtcgacttatctcgttttagccggt | *PikA* *3R SalI* |
| pHD1003 | gaagatctatgacgcaaaataagaaatcagt | *PikA* *5F Bgl II* |
| pHD1004 | ggcgattttgatatagctgttggaaatatagga | *PikA _H171A-_*1 |
| pHD1005 | tcctatatttccaacagctatatcaaaatcgcc | *PikA _H171A-_*2 |
| pHD1006 | gatatacatgttggagctataggagttatcag | *PikA _N174A-_*1 |
| pHD1007 | *ctgataactcctatagctccaacatgtatatc* | *PikA _N174A-_*2 |
| pHD1008 | *aaattagtaagaattgcttttgctggtagttt* | *PikA _D194A-_*1 |
| pHD1009 | aaactaccagcaaaagcaattcttactaattt | *PikA _D194A-_*2 |
| pHD1010 | cgcggatcccgactacaaagacgatgacgacaagatgacccagaacaaaaagagc | *Flag-PikA* *5F* *BamHI* |
| pHD1011 | ccggaattcttatctcgttttagccggtgt | *PikA* 3R *EcoRI* |
| pHD1012 | cgcggatccatggaatggcataagatacttaaagttac | *RvhD4* *5F BamHI* |
| pHD1013 | acgcgtcgacttactcattattttccggaacagttat | *RvhD4* *3R SalI* |
| pHD1014 | cgcggatccatggcttctgcatcaacttcta | *MTM1* *5F BamHI* |
| pHD1015 | acgcgtcgactcagaagtgagtttgcacatg | *MTM1* *3R SalI* |
| pHD1016 | gcttgtgcatagcagtgacg | MTM *_C375S_* -1 |
| pHD1017 | cgtcactgctatgcacaagc | MTM *_C375S_* -2 |
| pHD1018 | cgcggatccatggaagggtctaagacgtc | *BECN1 5F BamHI* |
| pHD1019 | cgcggatccgattacaaggatgacgacgataagatggaagggtctaagacgtc | *Flag-BECN1 5F BamHI* |
| pHD1020 | acgcgtcgactcatttgttataaaattgtgaggacacc | *BECN1 3R SalI* |
| pHD1021 | cgcggatccatggcagcctccg | *PLIPΔN68 5F BamHI* |
| pHD1022 | acgcgtcgacttagtttttggctgc | *PLIP 3R SalI* |
| pHD1023 | cgcggatccatggcggccgcagc | *Sac1p 5F BamHI* |
| pHD1024 | acgcgtcgactcagtctatcttttctttctggaccag | *Sac1p 3R SalI* |
| pHD1025 | tgaagatactaccttagggttcatcacta | *OmpB-5F* for qPCR |
| pHD1026 | accggcattaagcgtaaggtt | *OmpB-3R* for qPCR |
| pHD1027 | tgttgttcataacgctcac | TaqMan-MGB probe |

**Reference:**

1. Luo ZQ, Isberg RR. 2003. Multiple substrates of the Legionella pneumophila Dot/Icm system identified by interbacterial protein transfer. PNAS 101:841-846.

2. Karimova G, Pidoux J, Ullmann A, Ladant D. 1998. A bacterial two-hybrid system based on a reconstituted signal transduction pathway. PNAS 95:5752–5756.

3. Karimova G, Ullmann A, Ladant D. 2000. Bordetella pertussis adenylate cyclase toxin as a tool to analyze molecular interactions in a bacterial two-hybrid system. Int J Med Microbiol 290:441-5.

4. Song L, Xie Y, Li C, Wang L, He C, Zhang Y, Yuan J, Luo J, Liu I, Xiu Y, Li H, Gritsenko M, Nakayasu ES, Feng Y, Luo ZQ. 2021. The Legionella Effector SdjA Is a Bifunctional Enzyme That Distinctly Regulates Phosphoribosyl Ubiquitination. mBio 12:1-16.

5. Ma K, Shu R, Liu H, Ge J, Liu J, Lu Q, Fu J, Liu X, Qiu J. 2024. Legionella effectors SidC/SdcA ubiquitinate multiple small GTPases and SNARE proteins to promote phagosomal maturation. Cellular and Molecular Life Sciences 81.
